# Population-level respiratory virus–virus interactions, Puerto Rico, 2013–2023

**DOI:** 10.1016/j.ijid.2025.107878

**Published:** 2025-03-11

**Authors:** Zachary J. Madewell, Joshua M. Wong, Maile B. Thayer, Vanessa Rivera-Amill, Diego Sainz de la Peña, Jorge Bertrán Pasarell, Gabriela Paz-Bailey, Laura E. Adams, Yang Yang

**Affiliations:** 1 Division of Vector-Borne Diseases, Centers for Disease Control and Prevention, San Juan, Puerto Rico; 2 Ponce Health Sciences University/Ponce Research Institute, Ponce, Puerto Rico; 3 Auxilio Mutuo Hospital, San Juan, Puerto Rico; 4 Department of Statistics, Franklin College of Arts and Sciences, University of Georgia, Athens, GA, USA

**Keywords:** Co-infection, Viral interference, Acute respiratory infections, Pathogen interactions, Viral synergism

## Abstract

**Background:**

Understanding virus–virus interactions is important for evaluating disease transmission and severity. Positive interactions suggest concurrent circulation, while negative interactions indicate reduced transmission of one virus when another is prevalent. This study examines interactions among seven respiratory viruses using a Bayesian approach that accounts for seasonality and long-term trends.

**Methods:**

We analyzed data from 43,385 acute febrile illness cases in the Sentinel Enhanced Dengue Surveillance System in Puerto Rico (2013–2023). Viruses studied included influenza A (IAV), influenza B (IBV), respiratory syncytial virus (RSV), human parainfluenza viruses 1 and 3 (HPIV-1, HPIV-3), human adenovirus (HAdV), and human metapneumovirus (HMPV). Wavelet coherence analysis investigated synchronous or asynchronous viral co-variation, while a Bayesian hierarchical model estimated pairwise interactions.

**Results:**

Among 43,385 participants, 26.0% tested positive for at least one virus, with IAV (9.5%), HAdV (4.1%), RSV (3.6%), and IBV (3.6%) being most frequent. Coinfections occurred in 0.5% of cases, often involving HAdV. Wavelet coherence identified significant synchronization among RSV/HMPV, HPIV-1/HMPV, and other virus pairs, with minimal coherence during the COVID-19 pandemic. Bayesian modeling suggested five virus–virus associations: four positive (RSV/HPIV-3, HMPV/HPIV-1, IBV/HAdV, IBV/HMPV) and one negative (IAV/HAdV). However, when restricting the analysis to the prepandemic period, fewer associations remained statistically credible.

**Conclusion:**

Respiratory viruses in Puerto Rico demonstrate patterns of co-circulation that may reflect complex interactions, but these associations appear context-dependent. Findings highlight the need for continued surveillance to better understand virus–virus dynamics and their implications for public health interventions.

## Introduction

Respiratory viruses are a major public health concern, contributing significantly to global morbidity and mortality, particularly among vulnerable populations such as young children, the elderly, and individuals with underlying health conditions. These infections place a substantial burden on healthcare systems, leading to seasonal surges in hospitalizations, severe complications, and, in some cases, mortality. Infection by one virus could enhance or reduce infection and replication of a second virus, resulting in positive (additive or synergistic) or negative (antagonistic) interactions [1]. Mechanisms for these interactions range from cellular-level (e.g., blocking of cell surface receptors by influenza A virus [IAV], competition for resources like sialic acid by respiratory syncytial virus [RSV]), to host-level (impaired immune response caused by influenza leading to increased bacterial colonization), to population-level interactions (behavioral changes like self-isolation reducing transmission of other pathogens) [2,3]. Additionally, the initial viral infection can induce long-term specific immunity against the same virus or transient nonspecific immunity that offers broader protection against different, but related, viruses, particularly among taxonomically similar viruses [4].

Ecological factors also play an important role in shaping the circulation patterns of respiratory viruses. These include environmental conditions such as temperature and humidity, which can affect virus stability and transmission efficiency [5]. Seasonal behaviors, such as increased indoor crowding during colder months, can facilitate spread of certain viruses. Variations in host susceptibility due to prior exposure or vaccination campaigns further influence which viruses predominate in a given season [6]. For instance, influenza viruses often dominate during the winter, while adenoviruses and parainfluenza viruses circulate year-round but peak under specific conditions [7]. Positive virus–virus interactions describe a scenario where one virus creates conditions that aid another virus’s replication, transmission, or overall impact. Negative virus–virus interactions occur when a primary viral infection hinders the replication, transmission, or overall impact of a secondary viral infection [1].

Understanding both positive and negative interactions among viruses is useful for public health, as they can influence disease severity, transmission patterns, and effectiveness of interventions [1,8]. The increased availability of population-level etiological surveillance data—facilitated by advances in multiplex diagnostic testing and expanded testing during the COVID-19 pandemic—provides valuable insights into these interactions. However, limitations exist. Routine diagnostic practices often focus on one or two pathogens based on presenting symptoms and circulating trends, leading to biased incidence rates if the tested subpopulations differ significantly between viruses. Multiplex testing on representative clinical samples can mitigate this issue, but uncovering interactions requires large datasets collected over extended periods, often beyond the capacity of many studies [9]. Robust statistical methods are necessary to identify true virus–virus interactions while accounting for confounding factors such as seasonal and annual climatic variations, age-dependent contact patterns, and fluctuations in testing frequencies [10]. For instance, RSV peaks often coincide with colder weather, complicating the isolation of independent effects of temperature and co-circulation of other viruses on its transmission [11]. Similarly, co-circulation of human metapneumoviruses (HMPV) with influenza can result in more severe illness. Advanced analytic techniques, such as Bayesian hierarchical models and wavelet coherence analysis, can separate biological interactions from seasonal trends by explicitly modeling and adjusting for seasonality, long-term trends, and other confounders. These approaches isolate residual variation attributable to virus–virus interactions after accounting for known seasonal and environmental patterns.

To better understand the drivers and implications of virus–virus interactions, we use a novel Bayesian framework building on prior work for analyzing virus–virus interactions in public health surveillance data [8,10,12]. This approach is designed to evaluate population-level patterns of viral interactions while accounting for seasonal and long-term trends. However, it does not establish causality or distinguish biological mechanisms from shared environmental drivers. Here, we leverage this framework to evaluate positive and negative interactions among seven acute respiratory viruses: IAV, influenza B (IBV), RSV, human parainfluenza viruses 1 and 3 (HPIV-1, HPIV-3), human adenovirus (HAdV), and HMPV in Puerto Rico over an 11-year period.

## Methods

### Study population

We used data from the Sentinel Enhanced Dengue Surveillance System (SEDSS), an ongoing facility-based study in Puerto Rico that tracks the frequency and causes of acute febrile illness (AFI) [13,14]. Since 2012, SEDSS has included five sites: Centro Médico Episcopal San Lucas in Ponce, a tertiary acute care facility (2012-present), 2) Hospital Episcopal San Lucas—Guayama, a secondary acute care hospital (2013–2015), 3) Hospital de La Universidad de Puerto Rico in Carolina, another secondary acute care teaching hospital (2013–2015), 4) Centro de Emergencia y Medicina Integrada (CEMI), an outpatient acute care clinic in Ponce (2016-present), and 5) Auxilio Mutuo Hospital, a tertiary care facility in the San Juan Metro Area (2018-present).

### Study enrollment and data collection

SEDSS enrolls participants using convenience sampling. Potential participants were identified by triage nurses as any patient with an AFI defined by the presence of fever (≥38.0°C for temperatures measured orally, ≥37.5°C for temperatures measured rectally, and ≥38.5°C for temperatures measured axillary) at the time of triage or chief complaint of having a fever within the past 7 days. During the Zika epidemic (June 2016 to June 2018), patients were eligible if they presented with either rash and conjunctivitis, rash and arthralgia, or fever [15]. Starting in April 2020, in response to the COVID-19 pandemic, patients with cough or dyspnea within the last 14 days (with or without fever) were also eligible to better capture respiratory viruses [16]. No age groups were excluded; however, infants were eligible only if they presented to the hospital after their initial discharge from birth. After meeting the inclusion criteria and being informed about the study, participants provided written informed consent. In cases where patients were incapacitated at the time of triage due to acute illness, consent was sought after their stabilization.

SEDSS collects data via patient interviews and medical record reviews at enrollment and convalescence (~7–14 days later). The case investigation form (CIF) gathers demographics, comorbidities, and clinical features. The convalescent sample processing form (CSPF) echoes CIF data, adding the second specimen collection date and AFI severity indicators (hospitalizations, clinic visits). Initially paper-based (CIF/CSPF), data collection transitioned to electronic format in 2020 using REDCap on tablets [17].

### Sample collection and laboratory procedures

Blood, nasopharyngeal (NP), and oropharyngeal (OP) specimens were collected at enrollment from eligible participants. Additional blood samples (serum and whole blood) were also collected during the convalescent phase. Participation required providing at least one sample (blood or OP/NP swab). Molecular diagnostic testing of NP/OP specimens was done by reverse transcription–polymerase chain reaction on a panel of respiratory viruses including IAV and IBV, HAdV, RSV, HMPV, HPIV types 1 and 3, and SARS-CoV-2 as previously described [18,19]. All participants had molecular testing for dengue, chikungunya (December 2013 to April 2020), and Zika (December 2015 to April 2020) viruses for specimens collected within 7 days of symptom onset. Serologic testing was done by immunoglobulin M antibody capture enzyme-linked immunosorbent assay for anti-DENV, anti-CHIKV (December 2013 to March 2019), and anti-ZIKV (December 2015 to March 2019) antibodies for specimens collected >3 days after symptom onset [13].

### Statistical analyses

We first used wavelet coherence analysis to explore temporal co-variation patterns in virus incidence and prevalence across specific frequencies. We then calculated weighted Pearson’s correlation coefficients to evaluate the population-level covariation of respiratory virus infections, after removing seasonal and long-term trends. To more systematically adjust for confounding factors such as seasonality, site-specific variations, and demographic factors, we applied a multivariate Bayesian hierarchical model to analyze virus–virus interactions. In addition to removing seasonal and long-term trends, this model further accommodates sparse sampling, zero incidence, and autocorrelation. All analyses were done in R software, version 4.4.0 (R Foundation for Statistical Computing, Vienna, Austria). A comprehensive description of the statistical methods and sensitivity analyses is included in the Supplementary Methods.

## Results

### Study population and infection prevalence

**Figure 1.**
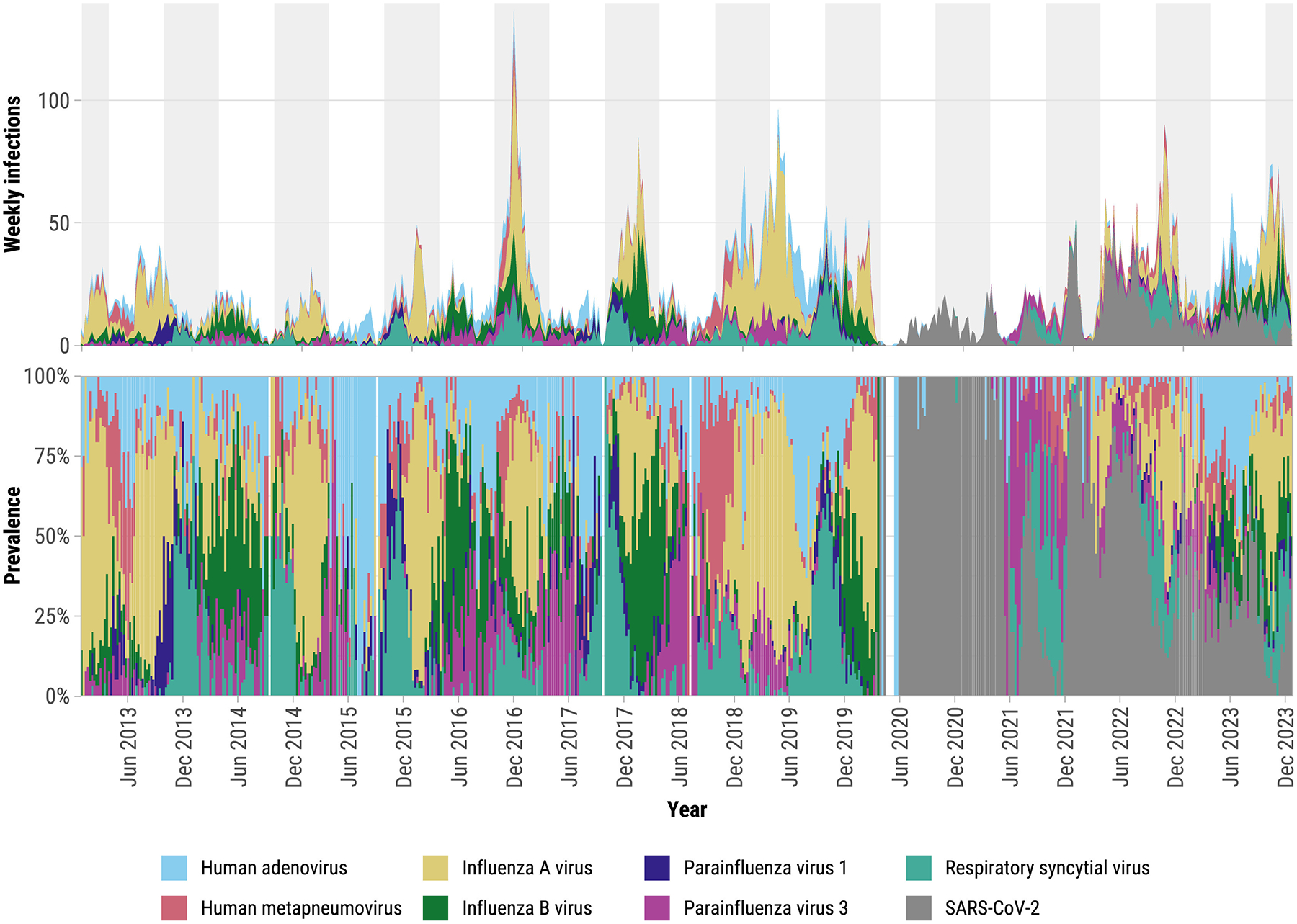
Laboratory-detected respiratory viral infections for each week from January 2013 to December 2023 (top) and relative prevalence of viruses (bottom), Sentinel Enhanced Dengue Surveillance System, Puerto Rico. Gray shading: October to March (winter respiratory virus season). Relative prevalence is calculated as the proportion of positive tests for each virus out of the total positive tests across all viruses detected in a given month.

Our dataset included 46,941 enrolled AFI cases from 2013 to 2023, of which 43,385 were tested for any of the viruses of interest, which was the denominator for all subsequent analyses. Of those cases, 52.8% were female, 53.9% were <18 years, and 99.6% were tested for all seven viruses (Table 1). One-tenth of participants had an arboviral infection including dengue (*n* = 869), chikungunya (*n* = 1619), and Zika (*n* = 1891). There were 11,288/43,385 (26.0%) participants who tested positive for any of the seven viruses studied and 208 (0.5%) were coinfected with more than one acute respiratory virus (Figure S3). The most frequent respiratory viral infections were IAV (*n* = 4127), HAdV (*n* = 1792), RSV (*n* = 1583), and IBV (*n* = 1547) (Figure 1, Table 1). The most frequent coinfections were HAdV with IAV (*n* = 40), RSV (*n* = 32), and HMPV (*n* = 22) (Figure S4). The proportion of participants testing positive for RSV, HPIV-1, HPIV-3, HAdV, and HMPV was highest among children <5, whereas IAV was high across all age groups (Figure S5). Monthly prevalences were significantly correlated between the two sexes for each virus, and the correlations were higher for IAV (*r* = 0.94), IBV (*r* = 0.92), and RSV (*r* = 0.92) than for other viruses (Figure S6).

### Wavelet coherence analysis

Wavelet coherence analysis revealed the strongest co-variation (dark red colors in Figure S7) for most virus pairs between 2015 and 2020, coinciding with the prepandemic period. Conversely, coherence became minimal during the COVID-19 pandemic (dark blue colors), reflecting disrupted transmission patterns. Significant co-variation indicates that the weekly prevalence of two viruses showed a consistent temporal relationship at specific frequencies. For example, RSV and HMPV (Figure S7, panel N) demonstrated synchronous co-variation at approximately 1-year cycles (between the 32- and 64-week marks on the y-axis), meaning their weekly prevalence patterns rose and fell together during these time periods. Similarly, RSV/HPIV-1 (panel L) and HPIV-1/HMPV (panel Q) also displayed synchronous co-variation at 1-year cycles, suggesting their fluctuations were aligned. HPIV-3/HMPV (panel S) and RSV/HAdV (panel O) showed synchronous co-variation at around 2-year cycles (64–128 weeks), while HPIV-3/HMPV showed a unique pattern with in-phase co-variation (fluctuating together) at 2-year cycles but anti-phase co-variation (fluctuating in opposite directions) at 1-year cycles. Several virus pairs, including IBV and HMPV (panel J), showed significant co-variation at shorter cycles (closer to the top of the y-axis), particularly around 2018–2019. In contrast, asynchronous co-variation, such as between IAV and HAdV (panel F), means that when one virus increased, the other tended to decrease over the same period.

### Partially adjusted correlations between viruses

**Figure 2.**
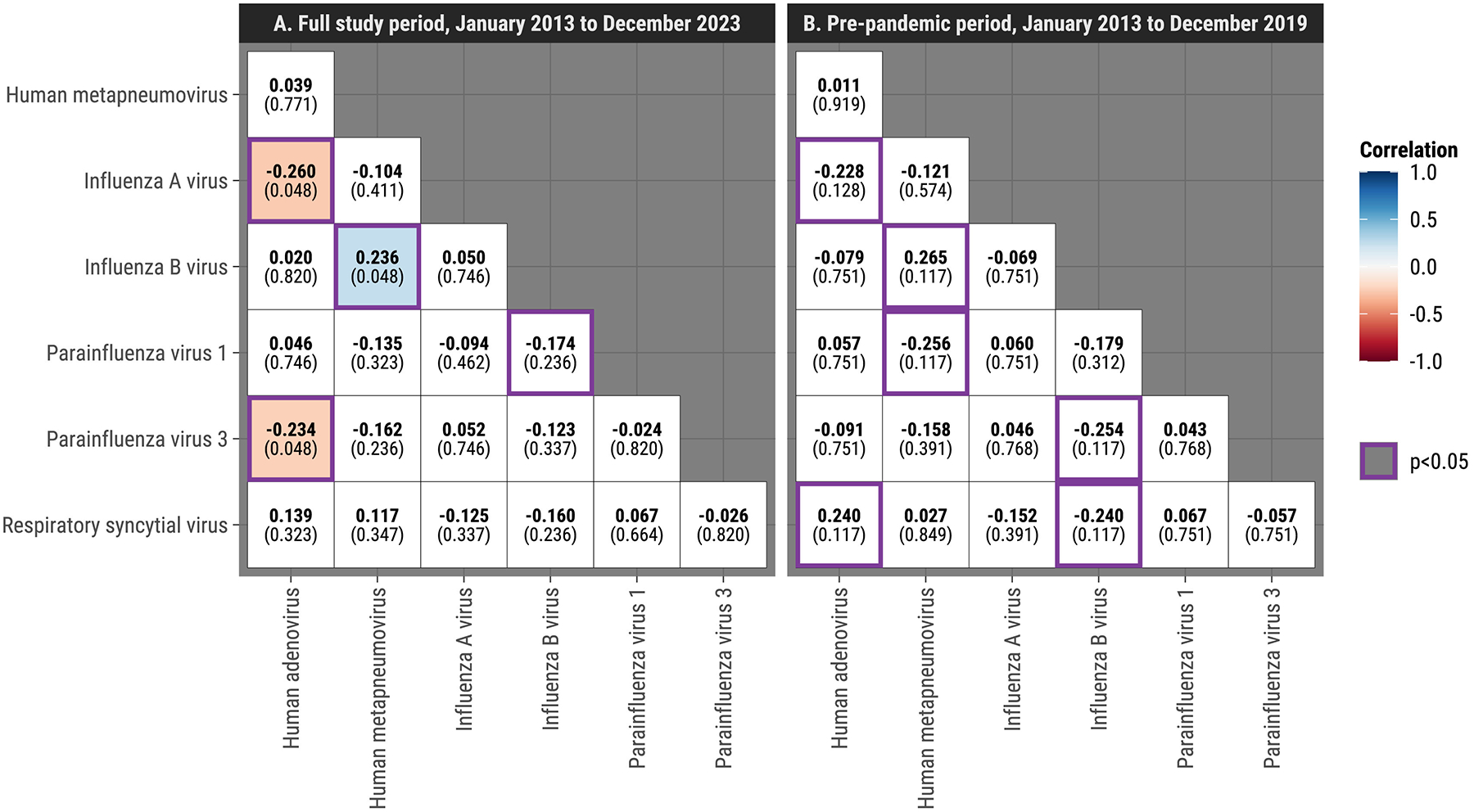
Weighted Pearson’s correlation coefficients of monthly prevalence for each pair of respiratory viruses, adjusted for seasonal and long-term trends, where weights correspond to the numbers of tests administered, Sentinel Enhanced Dengue Surveillance System, Puerto Rico. (a) Full study period (January 2013 to December 2023) and (b) prepandemic period (January 2013 to December 2019). *q*-values represent statistical significance after false discovery rate correction for multiple comparisons. Significant correlations (*q* ≤ 0.10) are shown in color, with blue indicating positive and red indicating negative correlations. For the prepandemic period, no virus pairs remained significant after adjusting for multiple comparisons by controlling the false discovery rate; therefore, virus pairs with nominal significance (*P* ≤ 0.05) are also shown with purple borders. Example interpretation: The positive correlation between IBV and HMPV suggests synchronous prevalence trends, meaning the two viruses tended to co-circulate during the study period. In contrast, the negative correlation between HAdV and HPIV-3 indicates asynchronous prevalence trends, where higher activity of one virus was associated with lower activity of the other. These associations should be interpreted cautiously, as they do not account for potential confounders like shared seasonality or testing patterns.

We evaluated weighted Pearson’s correlations between monthly prevalence of virus pairs, accounting for seasonal and long-term trends. When including all data from 2013 to 2023 and controlling for FDR ≤ 0.10 (Figure 2a), there were three significant virus–virus correlations: two negative (IAV/HAdV, HAdV/HPIV-3) and one positive (IBV/HMPV) indicating asynchronous and synchronous prevalence trends. Analyzing children separately yielded the same significant pairs and an additional negative association between HMPV and HPIV-3 (Figure S8). Restricting the analysis to the pre-COVID-19 pandemic period (2013–2019), six virus pairs were significant at *P* < 0.05 (four negative, two positive, Figure 2b); however, none of these pairs remained significant after adjusting for multiple comparisons.

Although these associations suggest potential virus–virus interactions, it is important to note that the correlation analysis cannot isolate biological interactions from confounding factors such as autocorrelation, shared seasonality, or testing variability. These limitations highlight the preliminary nature of this approach and the necessity of applying more robust methods. Our subsequent Bayesian hierarchical modeling addresses these confounders by explicitly accounting for seasonal patterns, long-term trends, and demographic factors, enabling a more refined investigation of potential virus–virus interactions.

### Seasonal and long-term trends

To identify seasonal patterns and long-term trends, we calculated the expected prevalence of each virus (Figures S1 and S2). Seasonal patterns were observed in influenza viruses, with IAV generally preceding IBV each year (Figure 1). IAV transmission typically occurred from November to March, while IBV peaked from April to June. Exceptions were noted in 2013 (IAV) and 2016–2017 (IBV) with extended circulation periods. Strong seasonality was observed for RSV, with epidemics typically starting between late October and early November and peaking in late November/early December. HAdV, HMPV, HPIV-1, and HPIV-3 circulated year-round but showed some seasonality. HAdV peaked in July, HPIV-1 in October, HPIV-3 in April to May, and HMPV in December to January. From March 2020 to April 2021, SARS-CoV-2 dominated respiratory infections, after which other respiratory viruses reemerged (Figure 1). HPIV-3 and RSV were the first respiratory viruses to reemerge in 2021, followed by IAV in 2022, and HAdV/IBV in 2023, demonstrating distinct temporal patterns post-COVID-19.

### Multivariate Bayesian hierarchical model

**Figure 3.**
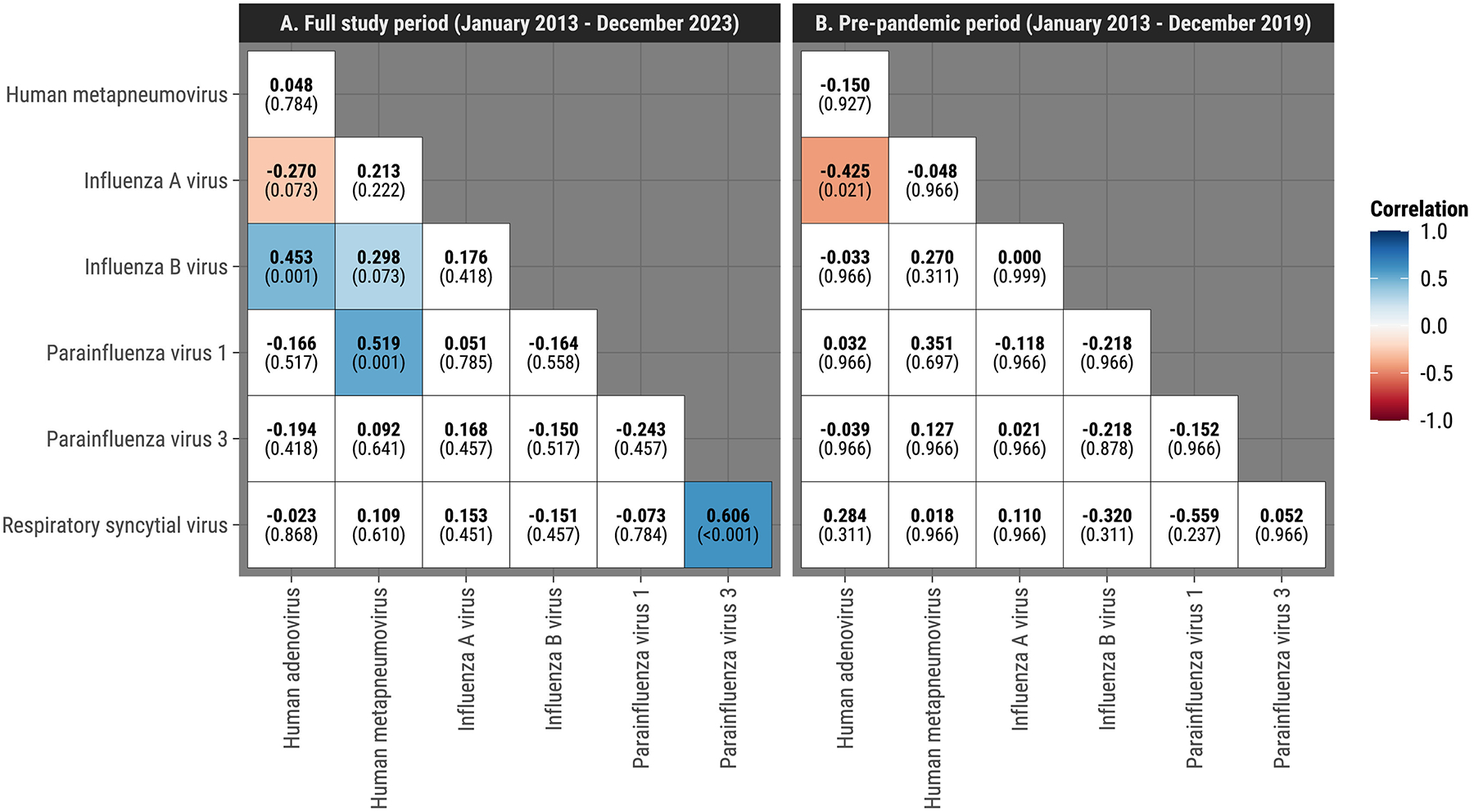
Bayesian hierarchical model correlation coefficients adjusting for age, sex, seasonality, changes in testing frequency, and autocorrelation, Sentinel Enhanced Dengue Surveillance System, Puerto Rico. (a) Full study period (January 2013 to December 2023) and (b) prepandemic period (January 2013 to December 2019). *q*-values represent statistical significance after false discovery rate correction for multiple comparisons. Significant correlations (*q* ≤ 0.10) are shown in color, with blue indicating positive and red indicating negative correlations. Example interpretation: A positive correlation between RSV and HPIV-3 (*ρ* = 0.61, 95% CrI: 0.36, 0.80, *q* ≤ 0.10) indicates that, after adjusting for confounding factors, detection of RSV was associated with a higher likelihood of detecting HPIV-3 during the same period, suggesting synchronous activity. Conversely, a negative correlation between IAV and HAdV (*ρ* = −0.27, 95% CrI: −0.48, −0.04, *q* ≤ 0.10) implies that higher prevalence of IAV was associated with lower prevalence of HAdV, reflecting asynchronous trends. These correlations account for demographic and temporal factors, providing insight into virus–virus interactions beyond simple seasonal overlap.

After removing seasonal and long-term trends, accounting for testing frequency, temporal autocorrelation, demographic factors (age group and sex), arboviral infection status, changes in testing practices during specific epidemic periods, and adjusting for multiple comparisons using *q*-values, Bayesian hierarchical models revealed four virus pairs with positive correlations and one with negative correlations, indicating increased and decreased likelihood of detection when one is present, respectively, with 0 and 1 indicating no and perfect correlation. The positive correlations were: RSV/HPIV-3 (*ρ* = 0.61, 95% CrI: 0.36, 0.80), HMPV/HPIV-1 (*ρ* = 0.52, 95% CrI: 0.22, 0.76), IBV/HAdV (*ρ* = 0.45, 95% CrI: 0.20, 0.65), and IBV/HMPV (*ρ* = 0.30, 95% CrI: 0.05, 0.53) (Figure 3a, Table S1). The negative correlation was between IAV and HAdV (*ρ* = −0.27, 95% CrI: −0.48, −0.04). IAV/HAdV and IBV/HMPV pairs showed consistent patterns of correlation in the same direction across both the partially adjusted and Bayesian multivariable model analyses, using *q*-values at the 0.10 level. In the subanalysis fitted solely on children, positive correlations were observed for IBV/HAdV (*ρ* = 0.46, 95% CrI: 0.20, 0.68), HMPV/HPIV-1 (*ρ* = 0.50, 95% CrI: 0.14, 0.79), and RSV/HPIV-3 (*ρ* = 0.66, 95% CrI: 0.41, 0.87) (Figure S9, Table S2). When restricting the analysis to the prepandemic period (2013–2019), only the negative correlation between IAV and HAdV remained statistically credible (*ρ* = −0.43, 95% CrI: −0.66, −0.14) (Figure 3b, Table S3).

## Discussion

Our findings suggest potential interactions among respiratory viruses in Puerto Rico over 11 years, which may influence disease severity, transmission, immune response, and vaccine effectiveness [1,3,8]. Identifying such correlations can inform public health strategies. For example, a positive correlation of 0.52 between HMPV and HPIV-1 suggests that when HMPV prevalence deviates from its expected seasonal trend, HPIV-1 prevalence tends to deviate in the same direction and by a moderate degree. Recognizing such correlations could allow hospitals to anticipate potential increases in certain infections, improving resource allocation for managing complications like croup and bronchiolitis. However, the observed interactions appear to be context-dependent rather than consistent drivers of virus transmission.

The impact of the COVID-19 pandemic on respiratory virus circulation was substantial, altering observed interaction patterns. From March 2020 to April 2021, SARS-CoV-2 became the dominant respiratory virus among SEDSS participants, reflecting trends in the continental U.S. [20]. This coincided with a sharp decline in IAV, IBV, RSV, HAdV, and other respiratory viruses following the implementation of stay-at-home orders and other nonpharmaceutical interventions [21,22]. These reductions persisted beyond the initial mitigation measures, likely due to changes in social interactions, testing behaviors, and potential viral interference [21]. Our analysis of the prepandemic period found that most virus–virus correlations identified in the full dataset did not remain statistically credible, suggesting that many observed interactions were influenced by pandemic-related disruptions. The only correlation that remained significant across both analyses was the negative association between IAV and HAdV, while other pairs, including IBV-HMPV and RSV-HPIV-3, were no longer significant in the prepandemic period. This finding suggests that some covariations may have been transient, potentially influenced by external factors such as testing practices, public health measures, or shifts in viral transmission dynamics. On the other hand, it could also be that the prepandemic data is underpowered to detect some interactions, especially the ones for which the magnitude and sign of the correlations remained consistent with those based on the whole study period, e.g., the pair of IBV and HMPV (Figure 2). These results underscore the need for caution in interpreting population-level correlations as definitive biological interactions.

Several studies from other countries have documented similar shifts in respiratory virus circulation due to viral interference with SARS-CoV-2. Nenna et al. [23] conducted a multicenter study in Italy and found that RSV hospitalizations peaked earlier than usual during the 2021–2022 season, coinciding with the relaxation of COVID-19 mitigation measures. Their findings suggest that SARS-CoV-2 may exert competitive pressure on RSV circulation, aligning with our observation of disrupted viral synchrony during the COVID-19 pandemic in Puerto Rico. Matera et al. [24] further reviewed global evidence of viral interference, highlighting how the emergence of SARS-CoV-2 altered epidemic patterns of influenza, RSV, and other respiratory viruses. Their review emphasizes the importance of considering virus–virus interactions when interpreting epidemiological trends, which is consistent with our findings using Bayesian hierarchical modeling. These studies reinforce the need for robust surveillance and modeling approaches to better understand how respiratory virus interactions shape epidemic patterns across different settings.

Consistent with previous research [10,25], positive correlations emerged between RSV/HPIV-3 and HMPV/HPIV-1, viruses commonly associated with lower respiratory tract infections in young children. HMPV/HPIV-1 also showed synchronous co-variation at approximately 1-year cycles in wavelet coherence analyses, suggesting their seasonal fluctuations might be driven by similar factors. Increased susceptibility in young children could result from frequent close contact in daycare and school settings, and limited prior exposure to these pathogens [26]. HPIVs, a leading cause of childhood lower respiratory tract infections, are slow-replicating viruses with extended infection times and a gradual decline in viral load [4]. This prolonged presence could increase the window of opportunity for encountering other respiratory viruses. The consistently high viral load observed for HPIV-3, regardless of coinfection status [27], further supports the notion of its prolonged presence within the host.

Our analysis confirms seasonal influenza patterns in Puerto Rico, with influenza A typically peaking from November to March and influenza B from April to June. While these peaks align with temperate regions, local tropical factors may cause slight variations in timing. Influenza A, which poses a higher risk of severe illness [28], peaks earlier in the season. This finding underscores the need for early vaccination efforts, as delaying vaccination could leave individuals vulnerable to influenza A, the more dangerous strain. Given that influenza B tends to extend later into the year, physicians should emphasize the importance of getting vaccinated as early as possible, ideally by the end of October, to provide protection during the critical influenza A season [29].

The negative correlation between IAV and HAdV in both partially adjusted and Bayesian analyses may reflect viral interference, potentially influenced by differences in replication cycles, tissue tropism, and seasonal variations, which can create a competitive environment unfavorable for simultaneous infections. Biological mechanisms, such as blocking cell surface receptors or competition for cellular resources, could underlie this interaction [1]. For example, neuraminidase expression in influenza A-infected cells removes sialic acid, preventing subsequent infections, and RSV replication is inhibited during co-infection with IAV due to competition for viral protein synthesis [30]. Ecological factors, including fluctuations in susceptible populations, age distribution, and testing practices, may also contribute to this negative correlation [25]. These findings likely reflect a combination of biological, ecological, and population-level dynamics rather than definitively confirming underlying biological mechanisms [8]. Host immune responses, particularly interferon induction, may mediate some interactions by providing transient protection against subsequent infections with different viruses [1]. Further research is needed to disentangle these drivers and better understand the observed dynamics.

Our study identified consistent positive correlations between IBV and HMPV across both Pearson correlation and Bayesian modeling, as well as between IBV and HAdV in Bayesian analyses. Although the mechanisms underlying these correlations require further investigation, IBV infection can damage respiratory epithelium and suppress the host’s immune response by downregulating antiviral proteins or interfering with antigen presentation, increasing susceptibility to secondary infections [31]. However, we acknowledge that our Bayesian approach, while adjusting for seasonality, testing patterns, and demographic factors, does not fully eliminate the possibility that shared environmental or epidemiological factors influence the observed associations. Differences in temperature, humidity, and human behavior (e.g., school attendance, holiday travel) could drive synchronized viral circulation independent of biological interactions [25].

Although the low individual-level co-infection rate among acute respiratory viruses (0.5%) in this study suggests single virus infections are most common, co-infections were observed and warrant further study. Studies have shown co-infections with respiratory viruses can lead to more severe illness, longer hospital stays, and worse clinical outcomes [32,33]. HAdV was involved in the most co-infections, consistent with other research [34,35].

Our study was subject to several limitations. First, it focused on seven respiratory viruses, excluding other pathogens like rhinovirus and bocavirus, which may influence co-infection patterns. Second, our data originated from hospital surveillance, capturing only symptomatic individuals seeking medical care. Respiratory viruses can present with mild or no symptoms, and these asymptomatic infections would not be captured through our healthcare-facility-based surveillance system, potentially affecting the observed correlations. Third, vaccination records were unavailable, which could affect infection and co-infection patterns. Fourth, we did not assess higher-order interactions between three or more viruses. Fifth, Bayesian modeling did not consider sensitivity and specificity of testing methods. Sixth, while our Bayesian hierarchical model accounts for many confounding factors, it does not fully isolate biological mechanisms from shared environmental drivers of viral circulation. Seasonal behaviors and climate factors may contribute to the observed correlations. Seventh, we could not determine the sequence of infections in individuals, and most virus pairs had too few co-infections (e.g., only 2 cases of RSV/IBV) to draw meaningful conclusions about within-host interactions. Future studies tracking repeated infections in the same individuals would be needed to distinguish biological interference from coincidental co-circulation. Notwithstanding these limitations, our study had a high testing rate (nearly 100%) for all seven viruses. The unique island context of our study potentially mitigates cross-contamination from external regions, strengthening the generalizability of the observed correlations between respiratory viruses.

The high testing rate for multiple viruses highlights the value of leveraging multiplex diagnostic data from hospitals and public health authorities to inform vaccine campaigns, resource allocation, and surveillance efforts. However, routine testing for viruses such as HPIV and HMPV is uncommon outside of high-resource hospitals with multiplex capabilities, as most facilities focus on influenza, COVID-19, and RSV. For hospitals with multiplex testing, using existing data to monitor trends in test positivity rates could help predict shifts in respiratory virus circulation—for example, a rise in HMPV cases may signal an impending increase in HPIV-1 prevalence.

This study in Puerto Rico identified potential correlations between respiratory viruses, providing preliminary insights for public health strategies. However, given the variability in observed associations across different periods, these findings should be interpreted with caution. While some interactions persisted across both full-period and prepandemic analyses, others appeared to be influenced by external factors such as the COVID-19 pandemic and shifts in testing and transmission dynamics. This highlights the need for continued surveillance to better understand the drivers of virus–virus co-circulation and their implications for public health.

Future research should continue using platforms like SEDSS to track respiratory virus interactions as new vaccines, such as the high-dose influenza vaccines for older adults and recently introduced RSV vaccines and monoclonals, enter the scene. The impact of widespread antiviral treatments on virus–virus dynamics also warrants investigation, as aggressive treatment for one virus (e.g., COVID-19 or influenza) may have positive or unanticipated effects on the prevalence and severity of other respiratory viruses. Exploring these effects will help refine prevention strategies and improve our understanding of respiratory pathogen interactions.

## Supplementary Material

upplementary material

Supplementary material associated with this article can be found, in the online version, at doi:10.1016/j.ijid.2025.107878.

## Figures and Tables

**Table 1:** Descriptive statistics of acute febrile illness cases who were tested for any of seven respiratory viruses, Sentinel Enhanced Dengue Surveillance System, Puerto Rico, 2013–2023.

Characteristics	Total *N* = 43,385 *n* (%)	<5 years *N* = 12,605 *n* (%)	5–17 years *N* = 10,758 *n* (%)	18–39 years *N* = 9797 *n* (%)	≥40 years *N* = 10,225 *n* (%)
Confirmed or probable arboviral infection					
Yes	4269 (9.8)	496 (3.9)	1190 (11.1)	1294 (13.2)	1289 (12.6)
No	39,116 (90.2)	12,109 (96.1)	9568 (88.9)	8503 (86.8)	8936 (87.4)
Tested for SARS-CoV-2 (*n* = 13,350)					
Positive	2043 (15.3)	200 (6.9)	215 (7.1)	618 (18.7)	1010 (24.5)
Negative	11,307 (84.7)	2703 (93.1)	2792 (92.9)	2692 (81.3)	3120 (75.5)
Tested for influenza A virus (*n* = 43,229)					
Positive	4127 (9.5)	831 (6.6)	1286 (12.0)	1103 (11.3)	907 (8.9)
Negative	39,102 (90.5)	11,761 (93.4)	9437 (88.0)	8644 (88.7)	9260 (91.1)
Tested for influenza B virus (*n* = 43,230)					
Positive	1547 (3.6)	241 (1.9)	734 (6.8)	311 (3.2)	261 (2.6)
Negative	41,683 (96.4)	12,352 (98.1)	9989 (93.2)	9436 (96.8)	9906 (97.4)
Tested for respiratory syncytial virus (*n* = 43,356)					
Positive	1583 (3.7)	1070 (8.5)	230 (2.1)	116 (1.2)	167 (1.6)
Negative	41,773 (96.3)	11,526 (91.5)	10,525 (97.9)	9673 (98.8)	10,049 (98.4)
Tested for human parainfluenza virus 1 (*n* = 43,354)					
Positive	447 (1.0)	277 (2.2)	113 (1.1)	31 (0.3)	26 (0.3)
Negative	42,907 (99.0)	12,318 (97.8)	10,641 (98.9)	9758 (99.7)	10,190 (99.7)
Tested for human parainfluenza virus 3 (*n* = 43,353)					
Positive	1036 (2.4)	655 (5.2)	137 (1.3)	82 (0.8)	162 (1.6)
Negative	42,317 (97.6)	11,940 (94.8)	10,617 (98.7)	9706 (99.2)	10,054 (98.4)
Tested for human adenovirus (*n* = 43,357)					
Positive	1792 (4.1)	1005 (8.0)	561 (5.2)	148 (1.5)	78 (0.8)
Negative	41,565 (95.9)	11,592 (92.0)	10,193 (94.8)	9641 (98.5)	10,139 (99.2)
Tested for human metapneumovirus (*n* = 43,357)					
Positive	963 (2.2)	463 (3.7)	220 (2.0)	114 (1.2)	166 (1.6)
Negative	42,394 (97.8)	12,134 (96.3)	10,534 (98.0)	9675 (98.8)	10,051 (98.4)
