# Supplementary material for: Population-level respiratory virus–virus interactions, Puerto Rico, 2013–2023
Source: Int J Infect Dis. Author manuscript; Available in PMC 2026 Mar 26. (PMC13019324; doi:10.1016/j.ijid.2025.107878)
Supplement: upplementary material [file NIHMS2158117-supplement-upplementary_material.docx]

**Supplementary Materials**

**Population-level respiratory virus-virus interactions, Puerto Rico, 2013–2023**

**Supplementary Methods**

**Figure S1.** The observed and expected prevalence of each virus before applying the multivariate Bayesian hierarchical model, January 2013 to December 2023, Sentinel Enhanced Dengue Surveillance System, Puerto Rico.

**Figure S2.** Sub-analysis restricted to the pre-pandemic period (January 2013 to December 2019) of observed and expected prevalence of each virus before applying the multivariate Bayesian hierarchical model Sentinel Enhanced Dengue Surveillance System, Puerto Rico.

**Figure S3.** Monthly numbers of laboratory-tested samples with a single respiratory viral infection, a viral co-infection, or determined to be negative of the seven viral infections studied from January 2013 to December 2023, Sentinel Enhanced Dengue Surveillance System, Puerto Rico.

**Figure S4.** Frequencies of acute respiratory viral coinfections, January 2013 to December 2023, Sentinel Enhanced Dengue Surveillance System, Puerto Rico.

**Figure S5.** The proportion of all tests for each virus that were positive and total number of infections by age group in years, January 2013 to December 2023, Sentinel Enhanced Dengue Surveillance System, Puerto Rico.

**Figure S6.** Monthly viral prevalence by sex from January 2013 to December 2023, Sentinel Enhanced Dengue Surveillance System, Puerto Rico.

**Figure S7.** Squared coherence in weekly prevalence between acute respiratory viruses, January 2013 to December 2023, Sentinel Enhanced Dengue Surveillance System, Puerto Rico.

**Figure S8.** Sub-analysis restricted to children of weighted Pearson’s correlation coefficients of monthly prevalence for each pair of respiratory viruses, adjusted for seasonal and long-term trends, where weights are the numbers of tests administered, Sentinel Enhanced Dengue Surveillance System, Puerto Rico, January 2013 to December 2023.

**Figure S9.** Bayesian hierarchical model correlation coefficients adjusting for age, sex, seasonality, changes in testing frequency, and autocorrelation, restricted to children <18 years, Sentinel Enhanced Dengue Surveillance System, Puerto Rico.

**Table S1.** Mean, standard deviation, and quantiles of the marginal posterior distribution for ρ, and convergence diagnostics for Bayesian hierarchical model for the total population, Sentinel Enhanced Dengue Surveillance System, Puerto Rico, January 2013 to December 2023.

**Table S2.** Mean, standard deviation, and quantiles of the marginal posterior distribution for ρ, and convergence diagnostics for Bayesian hierarchical model restricted to children <18 years old, Sentinel Enhanced Dengue Surveillance System, Puerto Rico, January 2013 to December 2023.

**Table S3.** Mean, standard deviation, and quantiles of the marginal posterior distribution for ρ, and convergence diagnostics for Bayesian hierarchical model for the total population restricted to the pre-COVID-19 pandemic period, Sentinel Enhanced Dengue Surveillance System, Puerto Rico, January 2013 to December 2019.

**Supplementary Methods**

*Wavelet coherence analysis*

We used wavelet coherence analysis to investigate the temporal dynamics of relationships between all respiratory viruses in this study [1]. By examining cyclical patterns in one virus’s incidence (e.g., IAV) relative to another virus (e.g., RSV), this technique reveals periods of synchronized or opposing cyclical patterns at specific frequencies. For example, significant co-variation might indicate that increases in RSV incidence during the winter are consistently followed by increases in HMPV incidence in subsequent weeks, suggesting a potential temporal relationship. For a more granular analysis, we aggregated data into weekly infection counts and calculated the prevalence of positive tests for each virus by dividing the number of infected patients by the total number of patients tested for each virus each week. Using squared coherency, wavelet coherence analysis identifies frequency bands where the two virus time series demonstrate statistically significant co-variation. Significance is established through Monte Carlo simulations using surrogate data (e.g., white noise) of the same length as the original virus incidence time series, allowing determination of the 95% confidence level for rejecting the null hypothesis of no co-variation at specific frequencies. We used the “biwavelet” package in R software for analyses [2].

*Pearson’s correlation*

For correlation analyses, we aggregated data into monthly infection counts for each virus to evaluate the population-level covariation patterns between viruses (132 total months). We calculated a measure of prevalence of positive tests for each virus as the number of infected patients over the total number of patients tested for each virus for each month. We then calculated weighted Pearson’s coefficients and 95% confidence intervals (CIs) to evaluate correlations in monthly infection prevalence between each of the 21 virus pairs, with weights corresponding to the number of tests administered. To account for potential seasonal and long-term trends in the data, we first decomposed the time series for each virus into seasonal, trend, and remainder components using Seasonal and Trend decomposition using Loess. We then used the remainder component, which represents the deseasonalized and detrended data, for the correlation analysis. The weighted Pearson’s correlation coefficients were calculated using the remainder components to ensure that the correlations reflect the true relationships between the virus pairs, independent of seasonal and long-term trends. The *p*-values were corrected for multiple comparisons using the Benjamini-Hochberg procedure [3], and false discovery rate (FDR)-adjusted *q* values were reported. Statistical significance was defined as *q* ≤ 0.10, that is, controlling the FDR at 10%. We also calculated weighted Pearson’s coefficients in monthly infection prevalence by sex for each virus.

*Multivariate Bayesian Hierarchical Model*

We employed a multivariate Bayesian hierarchical model framework, as previously described by Mair et al. [4, 5] and leveraged in our earlier publication [6], to analyze interactions among acute respiratory viruses. This approach addresses limitations of traditional methods by accommodating both dense and sparse sampling with zero-incidence events, while mitigating confounding effects such as intrinsic seasonality and long-term trends. By accounting for potential confounders like testing frequency, temporal autocorrelation, and seasonality, the model effectively disentangles true pathogen interactions from spurious correlations. It captures both within-year and between-year dependencies in infection risk, while simultaneously controlling for demographics and infection frequencies.

The model employs a two-stage approach, with the first stage involving preprocessing of observed monthly infection counts for each virus using mixed-effects logistic regression. This step adjusts for fixed effects, including age group, sex, any arboviral infection (dengue, chikungunya, or Zika), and changes in eligibility criteria during the Zika and COVID-19 epidemic periods. Specifically, eligibility was expanded during the Zika epidemic (June 2016–June 2018) to include patients presenting with either rash and conjunctivitis, rash and arthralgia, or fever, and during the COVID-19 pandemic (March 2020–April 2021) to include patients with cough or dyspnea within the last 14 days, with or without fever. To account for site-specific variations and different coverage years across the five SEDSS sites, a random intercept for each site was included. Seasonal effects were captured using harmonic functions, while long-term trends were modeled with polynomials of the year, selected based on minimizing the Akaike information criterion to achieve optimal balance between model complexity and capturing yearly and seasonal variations across viruses (Figures S1–S2). A quasi-binomial model was used to address overdispersion and excess zeros in the data. This preprocessing smooths observed monthly infection counts, removes potential confounding factors such as shifts in testing practices, and adjusts for reduced circulation of other respiratory viruses during epidemic periods. By comprehensively accounting for potential biases introduced by changing clinical practices and site-level differences, the model provides accurate estimates of seasonal trends while acknowledging the possibility of residual confounding.

We fit an overall generalized linear model for each virus, incorporating harmonic functions to capture seasonality and polynomials of year to account for long-term trends. To address the excessive number of zeros in the data, we used a quasi-binomial model that accounts for the zero-inflated nature of the data. Our model is specified as follows:

$$Z_{ymiv}\sim\text{quasi-Binomial}( \pi_{ymiv})$$

$$\log\left( \frac{\pi_{ymiv}}{1-\pi_{ymiv}} \right)=\beta_{v0}+\beta_{v1}+{\boldsymbol{\beta}_{\boldsymbol{v}\boldsymbol{2}}}^{\boldsymbol{'}}\boldsymbol{age}_{\boldsymbol{ymi}}\boldsymbol{+}\beta_{v3}{arbovirus}_{ymi}+\beta_{v4}{epoch\_Zika}_{ymi}+\beta_{v5}{epoch\_COVID}_{ymi}+{\boldsymbol{\beta}_{v6}}^{'}\boldsymbol{SEASON}_{m}+{\boldsymbol{\beta}_{v7}}^{'}\boldsymbol{POLY}_{y}+b_{site\left( i \right)}$$

Where $\pi_{ymiv}$ represents the test outcome (1=positive, 0=negative) for virus 𝑣 in the 𝑖^th^ sample during month 𝑚 of year 𝑦. The variable ${sex}_{ymi}$ indicates the sex of the individual, and ${\boldsymbol{\beta}_{\boldsymbol{v}\boldsymbol{2}}}^{\boldsymbol{'}}\boldsymbol{age}_{\boldsymbol{ymi}}$ is a vector of indicators for age groups. $\beta_{v3}{arbovirus}_{ymi}$ represents arboviral infection status (dengue, chikungunya, Zika). The terms $\beta_{v4}{epoch\_Zika}_{ymi}$ and $\beta_{v5}{epoch\_COVID}_{ymi}$ are binary indicators for changes in eligibility criteria during the Zika (2016–2018) and COVID-19 (2020–2021) periods, respectively. ${\boldsymbol{\beta}_{v5}}^{'}\boldsymbol{SEASON}_{m}$ describes seasonal oscillations using harmonic functions $\boldsymbol{(}sin\left( \frac{2\pi m}{12} \right), cos\left( \frac{2\pi m}{12} \right), sin\left( \frac{2^{2}\pi m}{12} \right),cos\left( \frac{2^{2}\pi m}{12} \right),sin\left( \frac{2^{3}\pi m}{12} \right),cos\left( \frac{2^{3}\pi m}{12} \right))$, and ${\boldsymbol{\beta}_{v6}}^{'}\boldsymbol{POLY}_{y}$ captures long-term trends through polynomial terms $(y, y^{2})$. $\beta_{v0}$, $\beta_{v1}$, $\beta_{v2}$, $\beta_{v3}$, $\beta_{v4}$, $\beta_{v5}$, $\beta_{v6}$, and $\beta_{v7}$ are the corresponding coefficients. $b_{site\left( i \right)}$ is a random intercept that captures the unique deviation for each site $i$ from the overall intercept and is assumed to be normally distributed with mean zero, i.e., $b_{site\left( i \right)}\sim N(0,\sigma_{site}^{2})$. We evaluated various model specifications using harmonic functions (up to eight cycles per year) and polynomials (up to quadratic) to capture seasonal and yearly variations. Selection was based on minimizing the Akaike information criteria, ensuring optimal balance between capturing yearly and seasonal variations while penalizing for model complexity, thus accommodating potential differences in terms and degrees among viruses. We calculated the expected prevalence of positive samples for each virus, month, and year as: $\hat{\pi}_{ymv}=\frac{1}{N_{ymv}}\sum_{i=1}^{N_{ymv}} \hat{\pi}_{ymiv}$, where $N_{ymv}$ is the total number of samples for each virus, month, and year, and $\hat{\pi}_{ymiv}$ is the model fitted probability for each sample.

In the second stage, we investigated pairwise interactions among the viruses and modeled each pair of viruses separately. We assume the observed prevalence ($\pi_{ymv}^{*}$) deviates from the expected prevalence ($\hat{\pi}_{ymv}$) estimated in the first stage by a virus-specific multiplicative random odds ratio (${OR}_{ymv}$):

$$\frac{\pi_{ymv}^{*}}{1-\pi_{ymv}^{*}}={OR}_{mtv}\times\frac{\hat{\pi}_{ymv}}{1-\hat{\pi}_{ymv}}$$

$${OR}_{ymv}=e^{\varphi_{ymv}-0.5\sigma_{v}^{2}}$$

The random OR (${OR}_{ymv}$) is obtained by exponentiating a normally distributed random effect ($\varphi_{mtv}$) with mean 0 and marginal variance $\sigma_{v}^{2}$. Subtracting $0.5\sigma_{v}^{2}$ from $\varphi_{ymv}$ ensures ${OR}_{ymv}$ has a mean 1. This framework allows us to impose flexible correlation structures on the random ORs to capture correlations between virus types across months and temporal correlations within each virus. The vector of random effects, $\varphi_{ymv}$, follows multivariate AR(1) structure, which captures temporal correlation between the random effects for consecutive months:

$\varphi_{y..}|\varphi_{(y-1)..}\sim\mathrm{MVN}(s_{v}\varphi_{\left( y-1 \right)..},\left[ \Omega\bigotimes\Lambda\right]^{-1})$.

Here, $\varphi_{y..}$ organizes virus type within each month depending on $\varphi_{(y-1)..}$ from the previous year, with $s_{v}<1$ as the autoregression coefficient. The precision matrix $\Omega=D-\lambda W$ captures the correlation of random effects between months via the neighborhood matrix $W$, where $w_{ij}=1$ if months $i$ and $j$ are neighbors and 0 otherwise. $\lambda$ is the smoothing parameter, set to 0.2. $D$ is a diagonal matrix with the diagonal element $D_{ii}=\sum_{j=1}^{12} w_{ij}$. We used a neighborhood dependency structure where the neighborhood for each month comprises four flanking months, two before and two after. Let $\Lambda={[\lambda_{ij}]}_{2\times2}$ be the precision matrix for the marginal bivariate normal distribution of $(\varphi_{ym1}, \varphi_{ym2})$. $\Lambda^{-1}$ represents the between-virus covariance matrix, and the corresponding correlation matrix is crucial for inferring virus-virus interactions at the population level. A common decomposition of the covariance matrix is: $\Lambda^{-1}=\Sigma\Gamma\Gamma^{T}\Sigma$

where $\Sigma$ is a diagonal matrix of standard deviations, and $\Gamma$ is the Cholesky decomposition of the correlation matrix, forming a lower-triangular matrix. We parameterize $\Gamma$ as $\Gamma=\left( \begin{matrix} 1 & 0 \\ \rho& 1 \end{matrix} \right)$ and $\Sigma=\left( \begin{matrix} \sigma_{1} & 0 \\ 0 & \sigma_{2} \end{matrix} \right)$. This setup ensures that the marginal variances and correlation coefficient between the two viruses are $\sigma_{1}^{2}$, $\sigma_{2}^{2}(1+\rho^{2})$ and $\rho/{\sqrt{1+\rho^{2}}}$, respectively. We report the posterior results of $\rho/{\sqrt{1+\rho^{2}}}$ to characterize the virus-virus interaction. Although the theoretical range of $\rho$ is ($-\infty,\infty$), a range of (-3, 3) for $\rho$ can effectively cover a range of (-0.95, 0.95) for the correlation coefficient. Therefore, we assign to $\rho$ a truncated normal prior, $N(0,1)I_{[-3,3]}$, ensuring that $\rho/{\sqrt{1+\rho^{2}}}$ is pulled away from 0 only when data contain a strong signal.

We can define the distribution of $N_{ymv}^{+}$, the number of positive tests for virus $v$ in month $m$ of year $y$, given $\pi_{ymv}^{*}$ and $N_{ymv}$. We used a zero-inflated binomial model for the monthly number of positive tests addressing both the excess zeros and the limited number of tests in certain months. Specifically, $N_{ymv}^{+}$ was modeled as:

$$N_{ymv}^{+}\sim Binomial(N_{ymv}, \pi_{ymv})$$

where $\pi_{myv}=\pi_{myv}^{*}U_{ymv}$ and $U_{ymv}\sim Bernoulli(p_{v})$. $U_{mtv}$ represents an additional Bernoulli random variable introduced to account for zero-inflation, with $p_{v}$ denoting the associated probability, which varies by virus. When $U_{mtv}=0$, $N_{ymv}^{+}=0$; when $U_{mtv}=0$, $N_{ymv}^{+}\sim Binomial(N_{ymv}, \pi_{myv}^{*})$. Consequently, $N_{ymv}^{+}$ has an extra probability of $1-p_{v}$ to equal 0.

Markov chain Monte Carlo simulations were used to estimate credible intervals for correlation coefficients (*ρ*), derived from the covariance matrices of the model’s random effects, capturing the strength and directionality of virus-virus interactions. Bayesian Z-scores, representing the ratio of posterior means to posterior standard deviations, were computed from posterior samples to derive asymptotic two-sided *p*-values. These *p*-values were further adjusted to control the false discovery rate (FDR), and *q*-values <0.10 were considered statistical evidence of interactions. Credible intervals for $\rho/{\sqrt{1+\rho^{2}}}$ were determined from posterior sample quantiles, with the 95% and 90% credible intervals corresponding to the (2.5%, 97.5%) and (5%, 95%) sample quantiles, respectively. The analysis used five chains, each comprising 100,000 iterations, with the first 10,000 iterations discarded as burn-in, and 900 thinned draws retained from each chain for further analysis. Convergence of the chains was assessed using the Gelman-Rubin statistic [7], effective sample size, and effective number of parameters. Analyses were conducted for the total population and children <18 years separately, given their vulnerability to lower respiratory tract infections and potentially distinct disease patterns. A sub-analysis was also performed for pre-pandemic years (2013–2019) to evaluate virus-virus interactions before disruptions caused by the COVID-19 pandemic. Models were fitted using the R2jags package in R statistical software (R Development Core Team, Vienna, Austria) [8, 9].

SARS-CoV-2 testing data were included in descriptive analyses (e.g., Table 1 and Figure 1) to provide context on respiratory virus testing and the impact of the COVID-19 pandemic on circulation patterns. However, SARS-CoV-2 was excluded from wavelet coherence, Pearson correlation, and Bayesian hierarchical model analyses due to its limited data availability (2020 onward) and the significant pandemic-related disruptions to respiratory virus dynamics. These factors reduced the statistical power to estimate interactions and complicated efforts to account for seasonality and long-term trends. The Bayesian hierarchical model, in particular, relies on several years of consistent data for robust estimates of virus-virus interactions.

**References**

1. Torrence C, Compo GP. A practical guide to wavelet analysis. Bulletin of the American Meteorological society. 1998;79(1):61-78.

2. Gouhier TC, Grinsted A, Simko V, Gouhier MTC, Rcpp L. Package ‘biwavelet’. Spectrum. 2013;24:2093-102.

3. Benjamini Y, Drai D, Elmer G, Kafkafi N, Golani I. Controlling the false discovery rate in behavior genetics research. Behavioural brain research. 2001;125(1-2):279-84.

4. Nickbakhsh S, Mair C, Matthews L, Reeve R, Johnson PCD, Thorburn F, et al. Virus-virus interactions impact the population dynamics of influenza and the common cold. Proc Natl Acad Sci U S A. 2019;116(52):27142-50.

5. Mair C, Nickbakhsh S, Reeve R, McMenamin J, Reynolds A, Gunson RN, et al. Estimation of temporal covariances in pathogen dynamics using Bayesian multivariate autoregressive models. PLoS Comput Biol. 2019;15(12):e1007492.

6. Madewell ZJ, Wang L-P, Dean NE, Zhang H-Y, Wang Y-F, Zhang X-A, et al. Interactions among acute respiratory viruses in Beijing, Chongqing, Guangzhou, and Shanghai, China, 2009–2019. Influenza and Other Respiratory Viruses. 2023;17(11):e13212.

7. Gelman A, Rubin DB. Inference from iterative simulation using multiple sequences. Statistical science. 1992;7(4):457-72.

8. Plummer M, editor JAGS: A program for analysis of Bayesian graphical models using Gibbs sampling. Proceedings of the 3rd international workshop on distributed statistical computing; 2003: Vienna, Austria.

9. R Core Team R. R: A language and environment for statistical computing. R foundation for statistical computing Vienna, Austria; 2013.

**Figure S1**. The observed and expected prevalence of each virus before applying the multivariate Bayesian hierarchical model, January 2013 to December 2023, Sentinel Enhanced Dengue Surveillance System, Puerto Rico. The expected prevalence was calculated by fitting generalized linear models for each virus with harmonic functions to account for seasonality and polynomials to account for long-term trends, while adjusting for sex, age group, arbovirus infection, changes in eligibility during Zika and COVID-19, and hospital site. Prevalence was the number of infected patients over the total number of patients tested for each virus for each month.


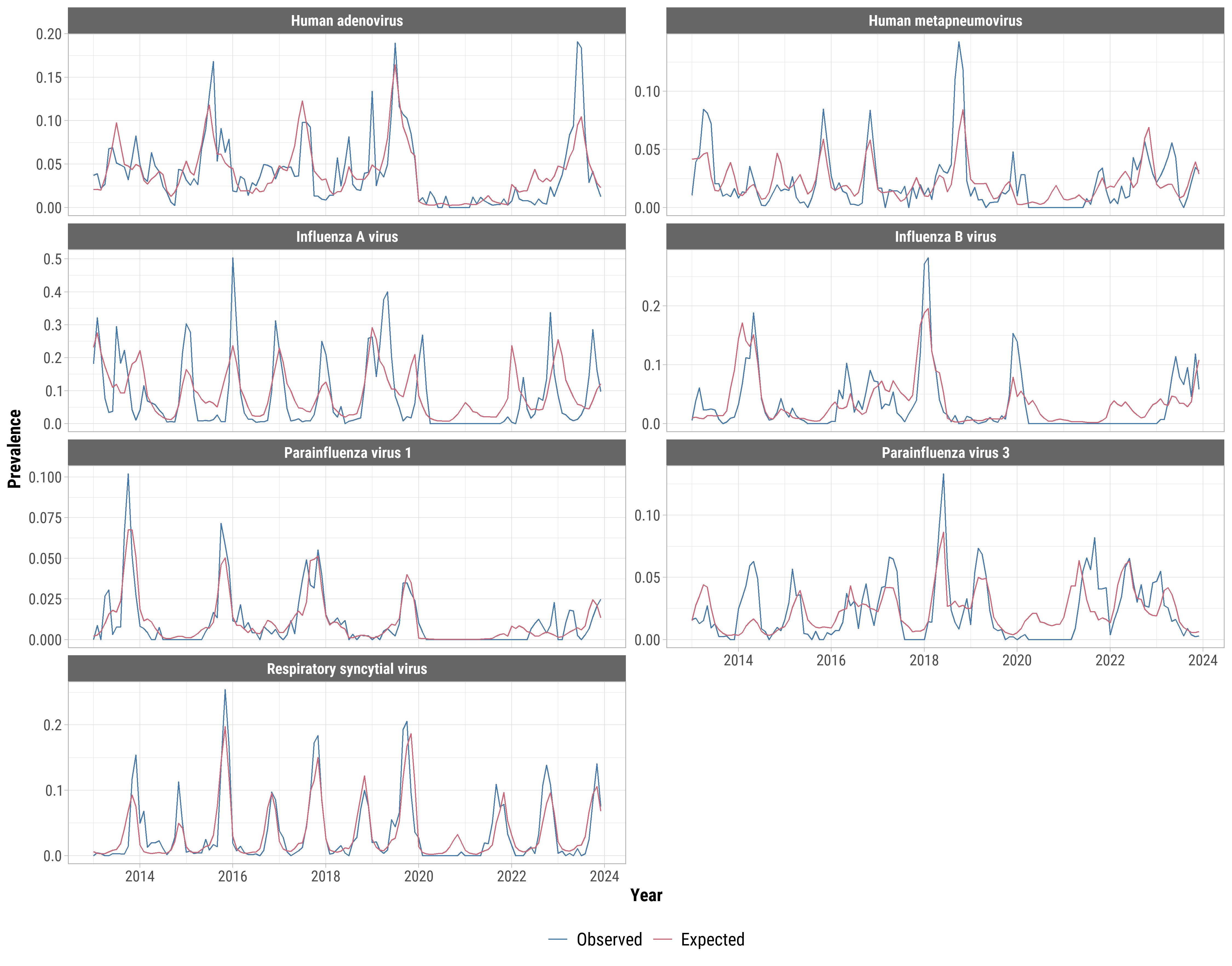


**Figure S2**. Sub-analysis restricted to the pre-pandemic period (January 2013 to December 2019) of observed and expected prevalence of each virus before applying the multivariate Bayesian hierarchical model Sentinel Enhanced Dengue Surveillance System, Puerto Rico. The expected prevalence was calculated by fitting generalized linear models for each virus with harmonic functions to account for seasonality and polynomials to account for long-term trends, while adjusting for sex, age group, arbovirus infection, changes in eligibility during Zika, and hospital site. Prevalence was the number of infected patients over the total number of patients tested for each virus for each month.


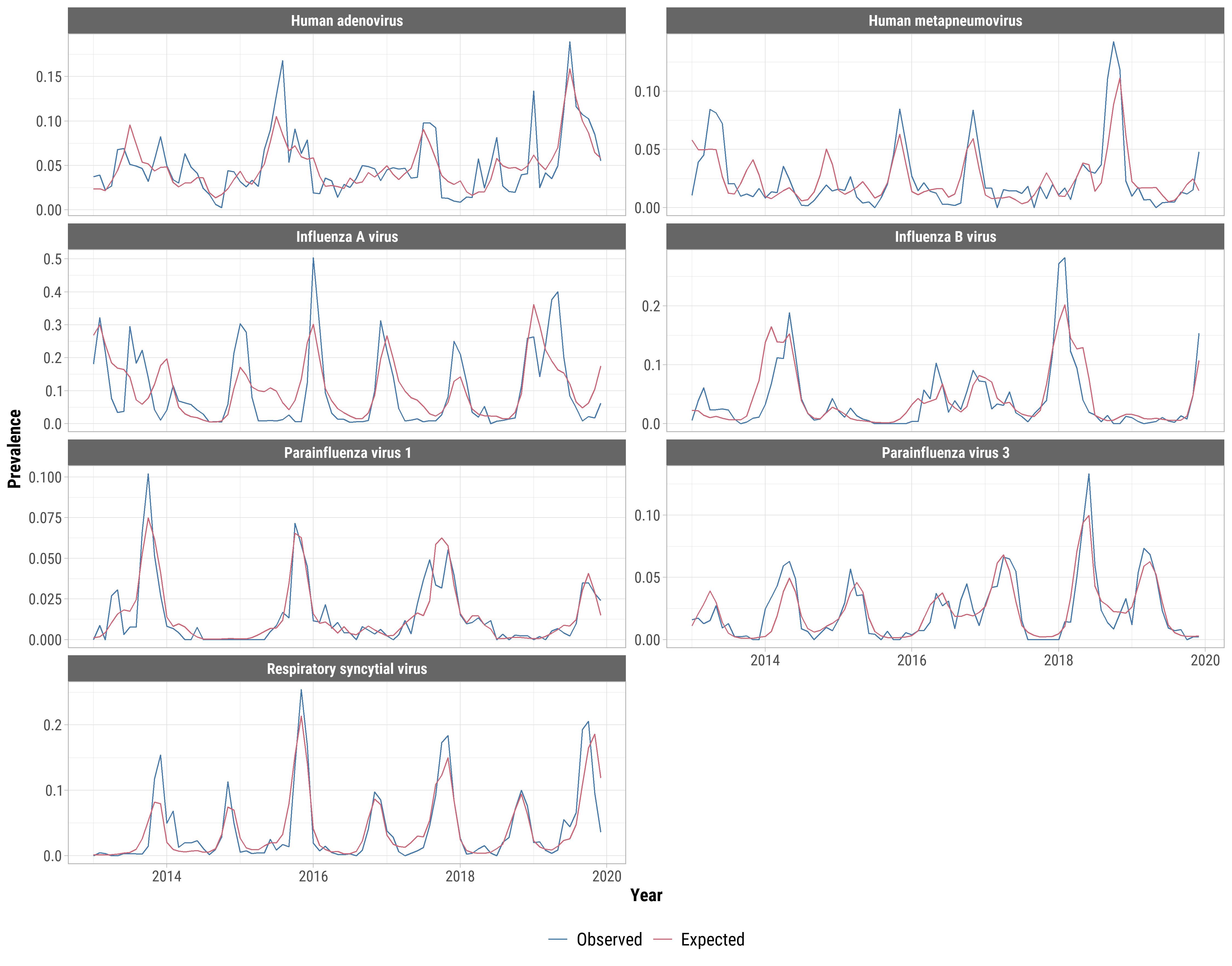


**Figure S3.** Weekly numbers of laboratory-tested samples with a single respiratory viral infection, a viral co-infection, or determined to be negative of the seven viral infections studied (influenza A, influenza B, respiratory syncytial virus, human parainfluenza virus types 1 and 3, human adenovirus, and human metapneumovirus) from January 2013 to December 2023, Sentinel Enhanced Dengue Surveillance System, Puerto Rico.

**
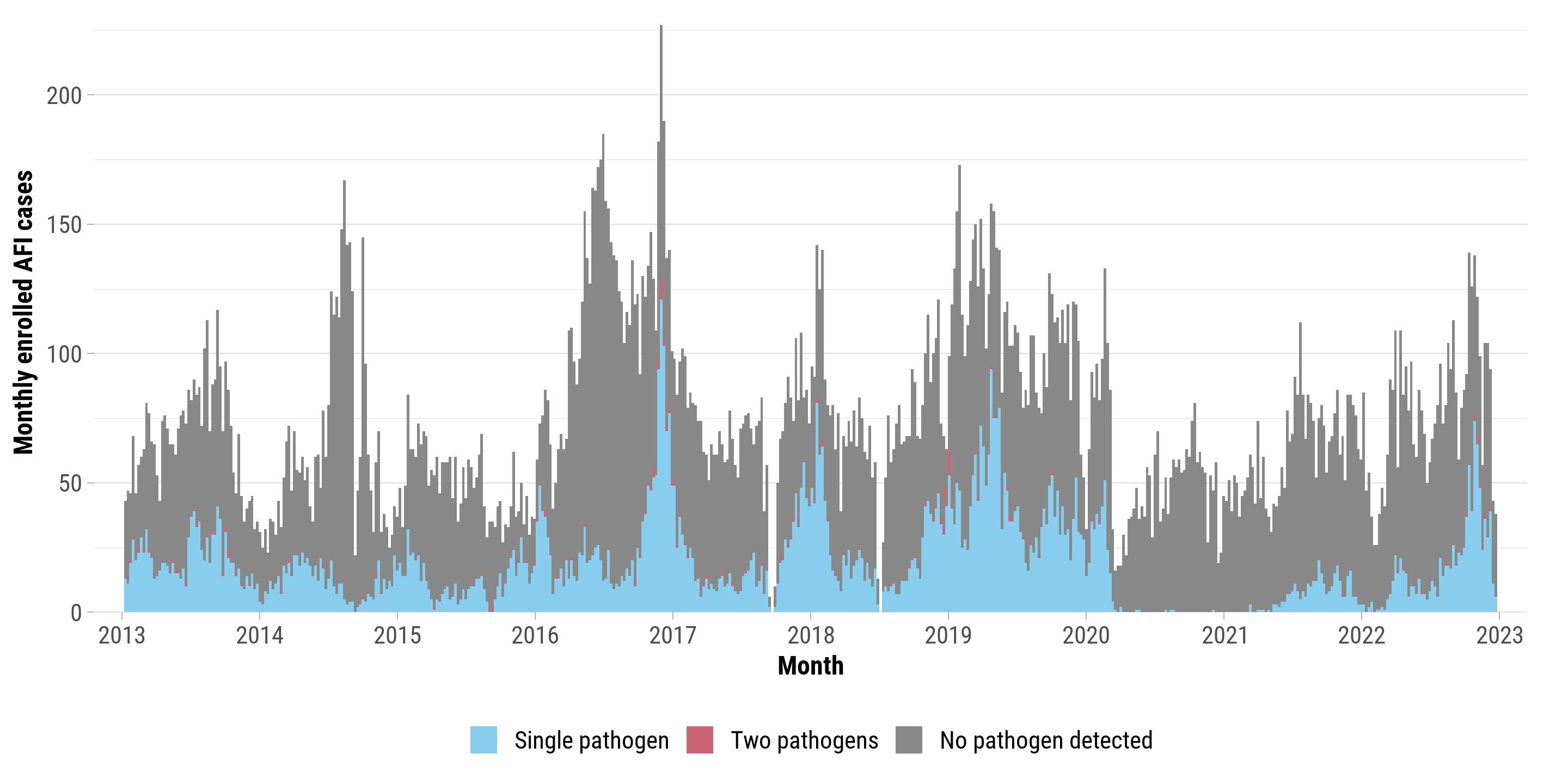
**

**Figure S4**. Frequencies of acute respiratory viral coinfections, January 2013 to December 2023, Sentinel Enhanced Dengue Surveillance System, Puerto Rico.


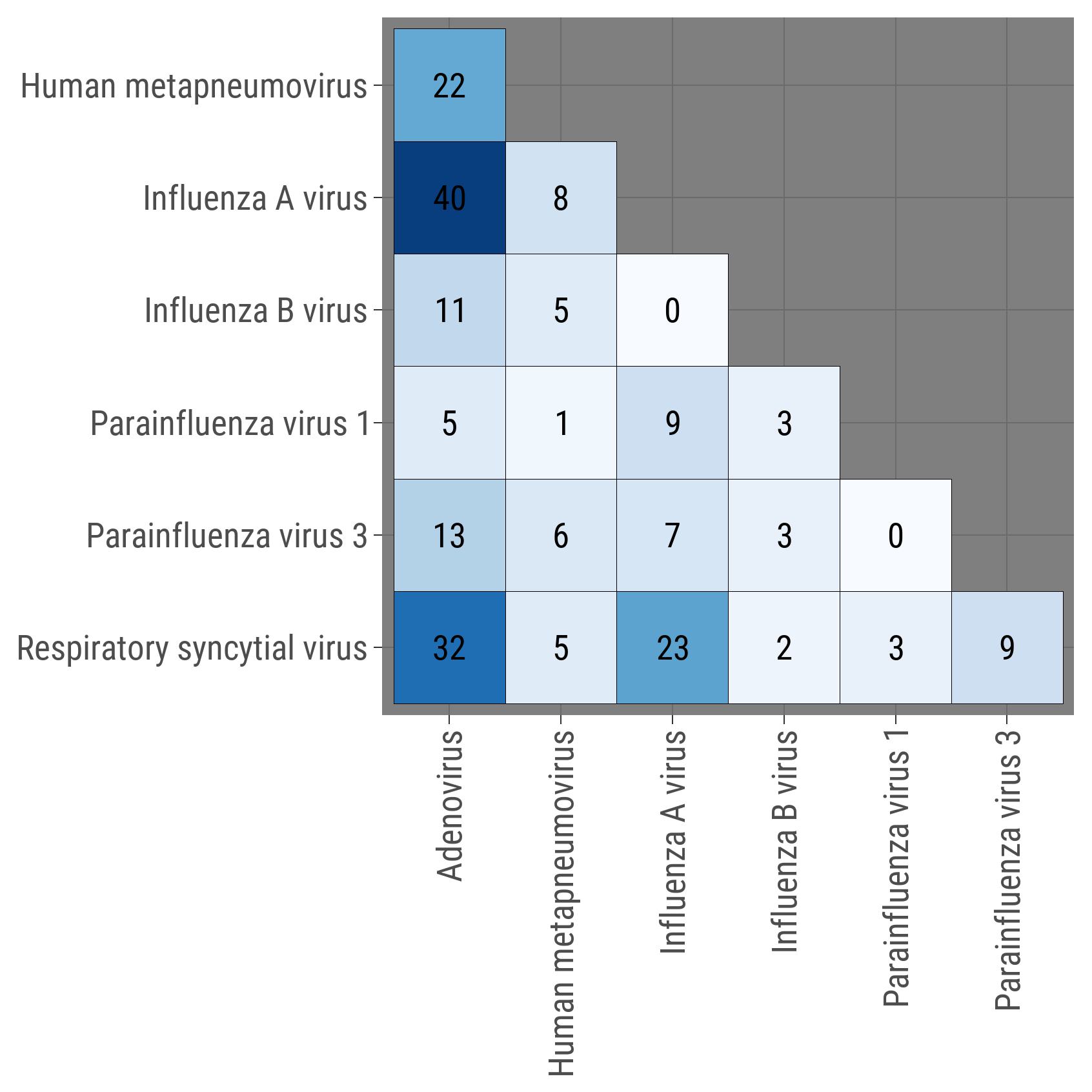


**Figure S5**. The proportion of all tests for each virus that were positive and total number of infections by age group in years, January 2013 to December 2023, Sentinel Enhanced Dengue Surveillance System, Puerto Rico. Larger circles represent a greater proportion of positive tests and darker colors represent greater numbers of infections.


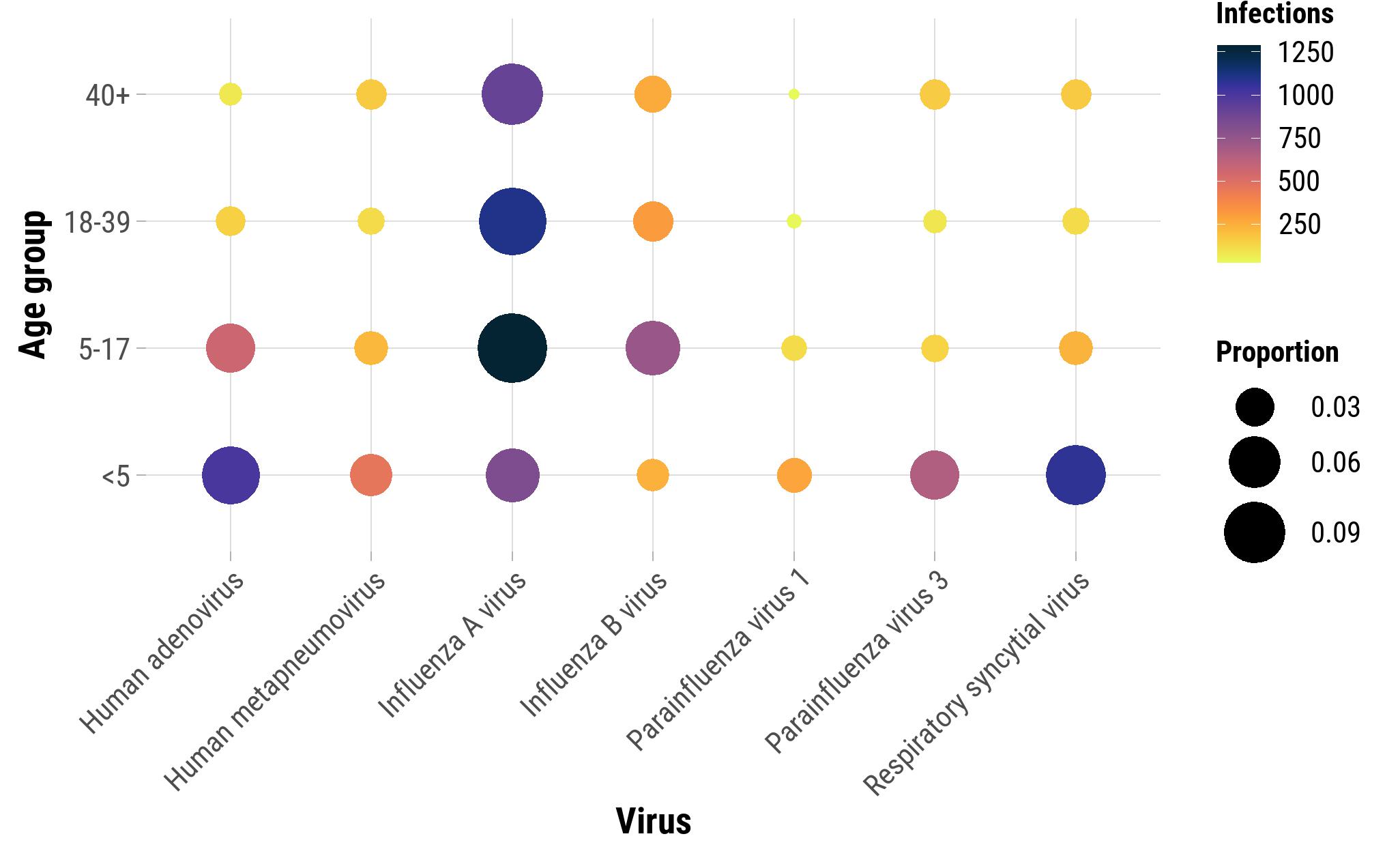


**Figure S6**. Monthly viral prevalence by sex from January 2013 to December 2023, Sentinel Enhanced Dengue Surveillance System, Puerto Rico. Weighted Pearson’s coefficients in monthly infection prevalence between the two sexes for each virus in each city and 95% confidence intervals are shown, with weights corresponding to the number of tests administered by city. Prevalence was the number of infected patients over the total number of patients tested for each virus for each month.
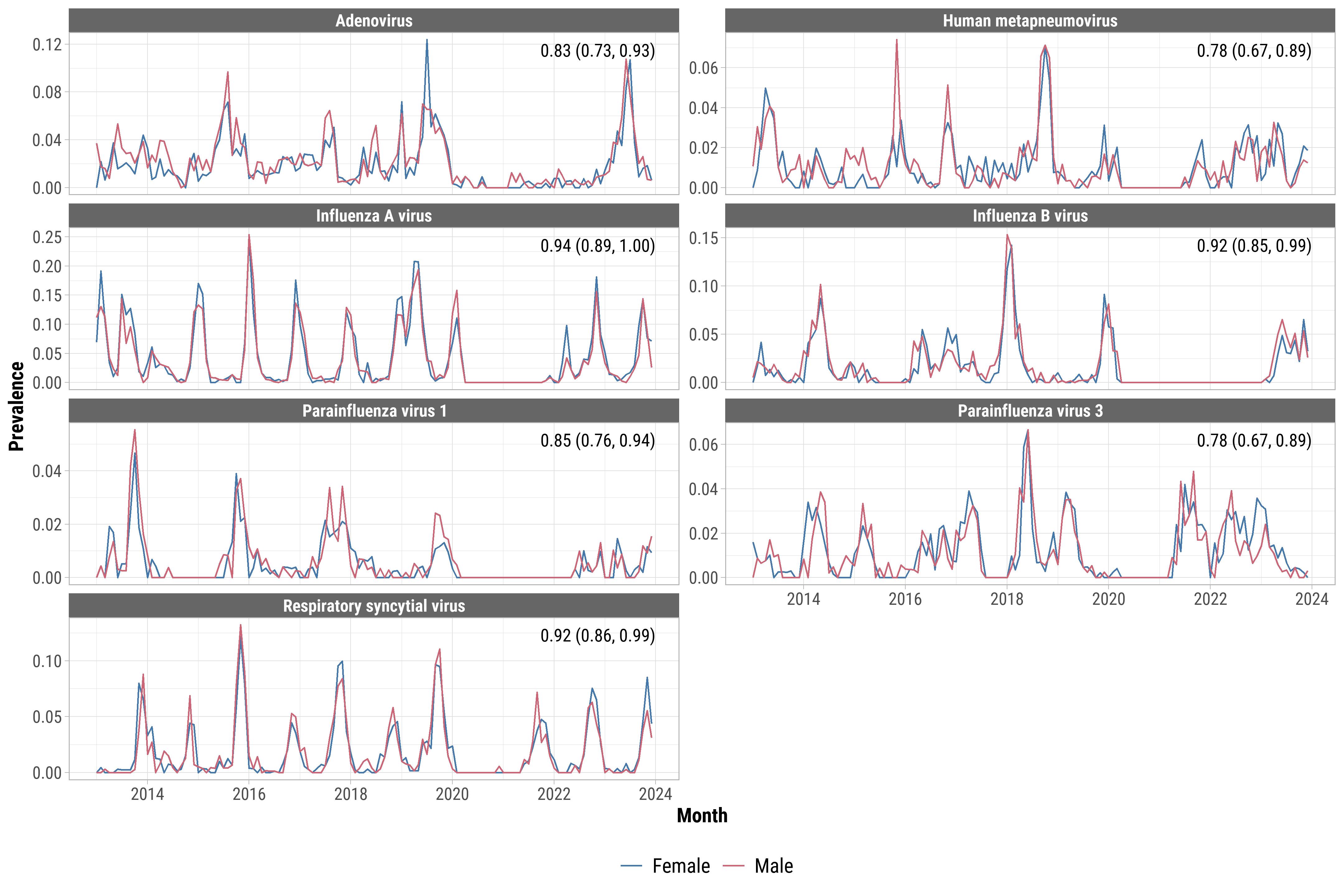


**Figure S7.** Squared coherence in weekly prevalence between acute respiratory viruses, January 2013 to December 2023, Sentinel Enhanced Dengue Surveillance System, Puerto Rico. The y-axis represents time periods in weeks, with lower frequencies (longer cycles) at the bottom and higher frequencies (shorter cycles) at the top. Warmer colors (red) indicate stronger coherence (i.e., greater co-variation) between virus pairs, whereas cooler colors (blue) indicate weaker coherence. Significant coherence regions are enclosed in black contours, and arrows indicate the phase relationship, with rightward arrows showing in-phase relationships and leftward arrows showing anti-phase relationships. For example, for RSV and HPIV-1 (Panel L), strong coherence was observed between 2015 and 2020, particularly at 32–64-week cycles (one-year periodicity) and 64–128-week cycles (approximately two-year periodicity). The arrows within the significant regions (outlined in black) show the phase relationship: rightward arrows indicate that RSV and HPIV-1 were in phase, meaning their prevalence trends rose and fell synchronously during these periods. However, during the COVID-19 pandemic (2020–2022), coherence weakened substantially, as evidenced by the predominance of cooler colors. This reflects the disrupted transmission patterns of respiratory viruses caused by pandemic-related interventions, such as mask-wearing, school closures, and reduced social contact.

| **A.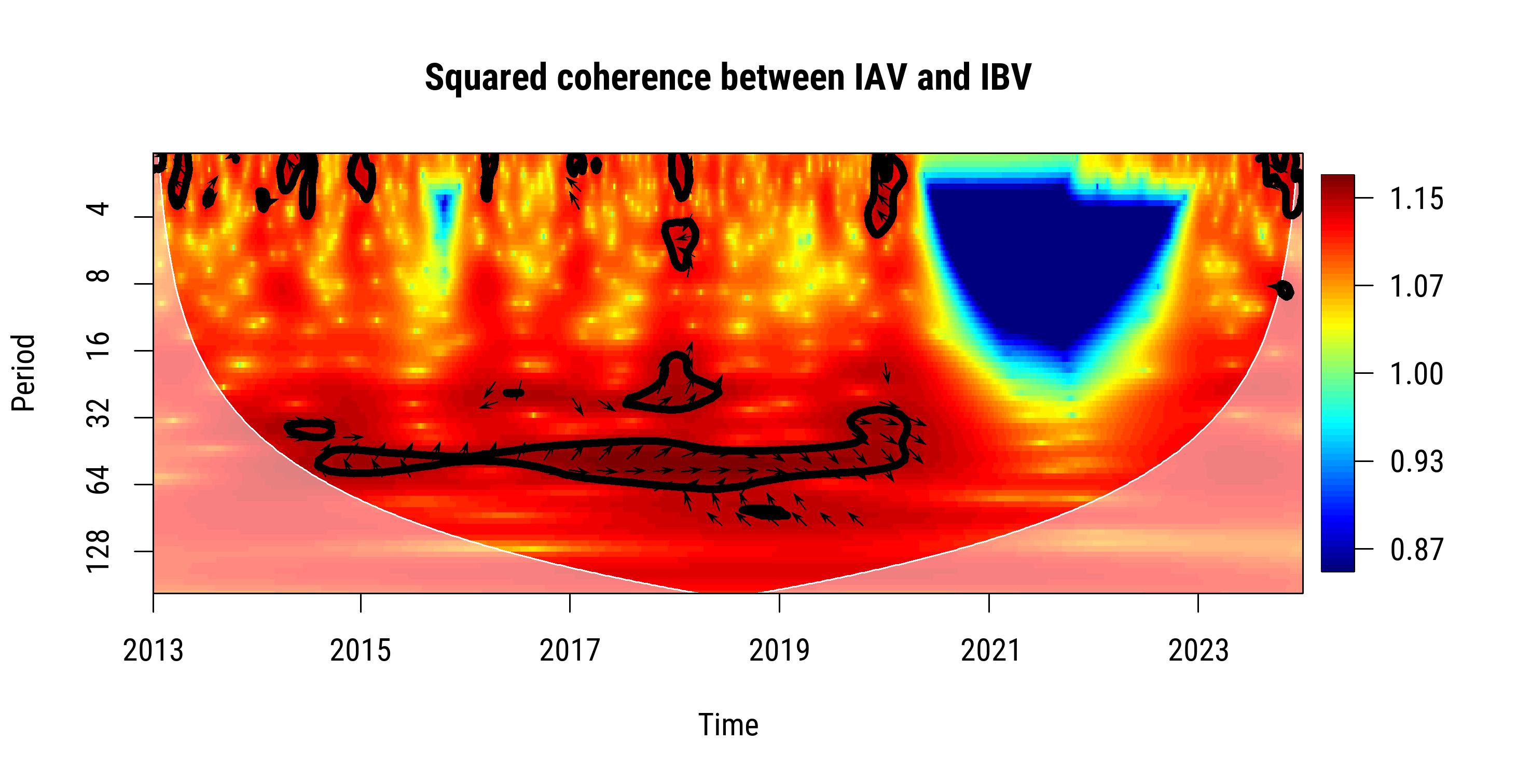** | **B.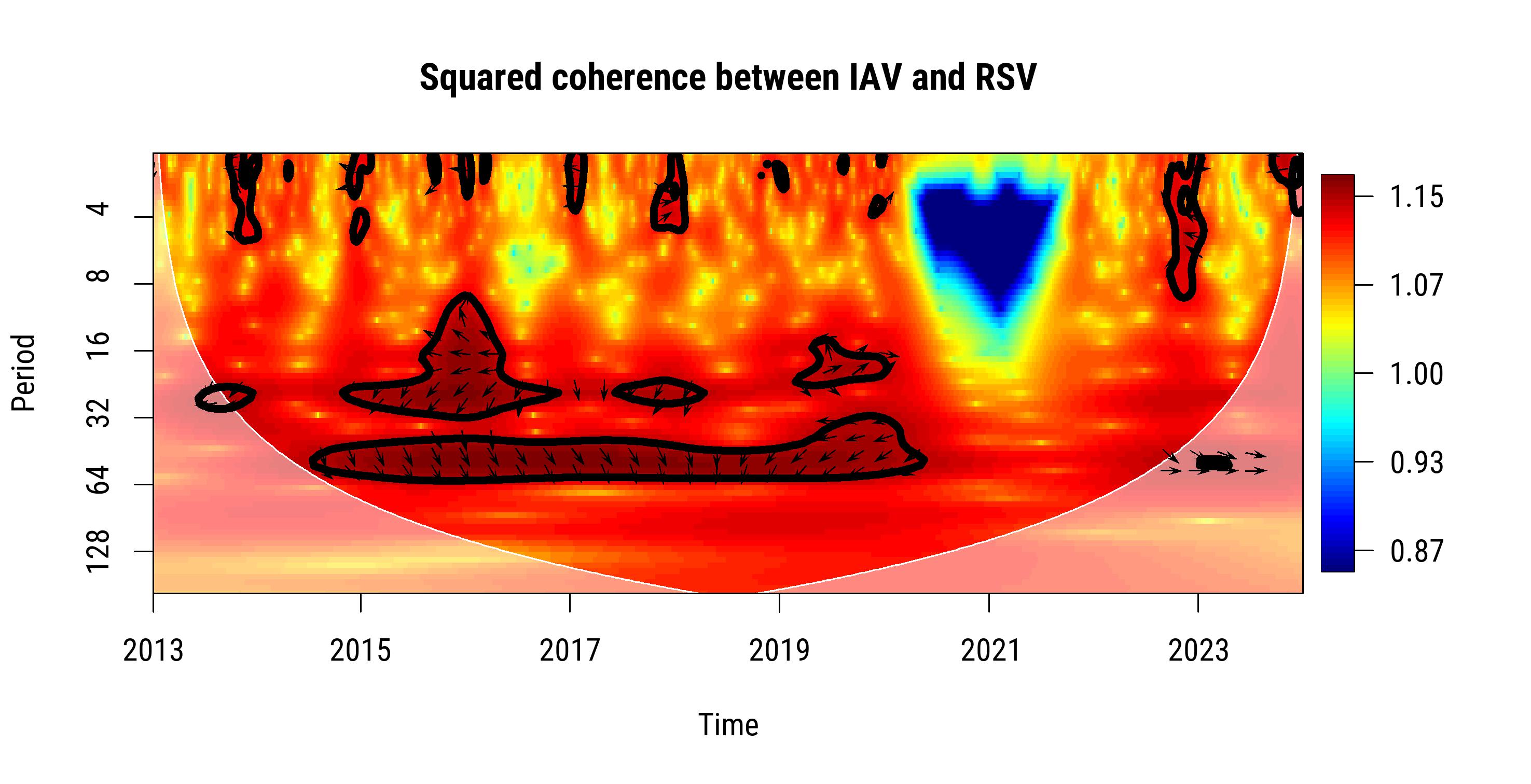** |
| --- | --- |
| **C.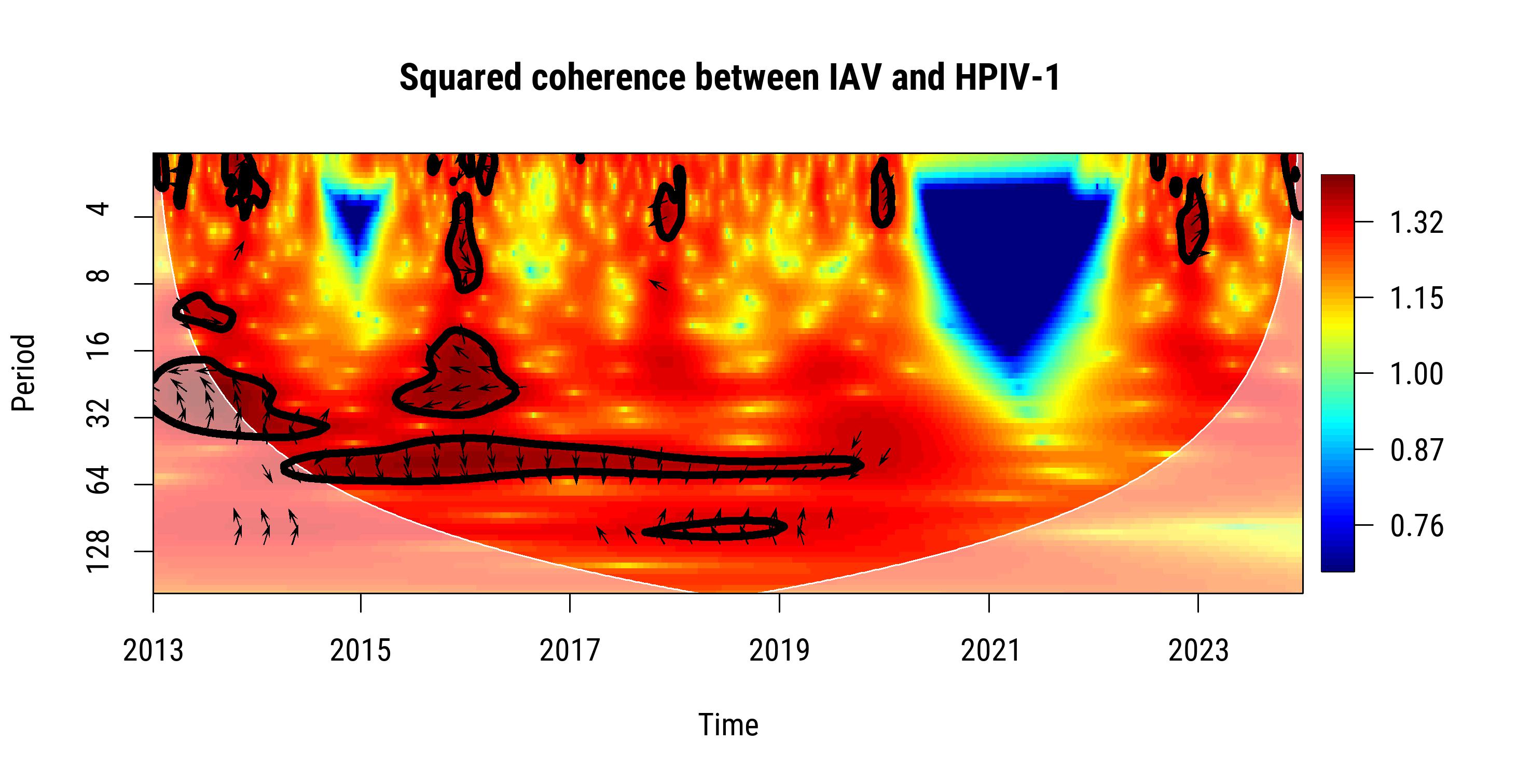** | **D.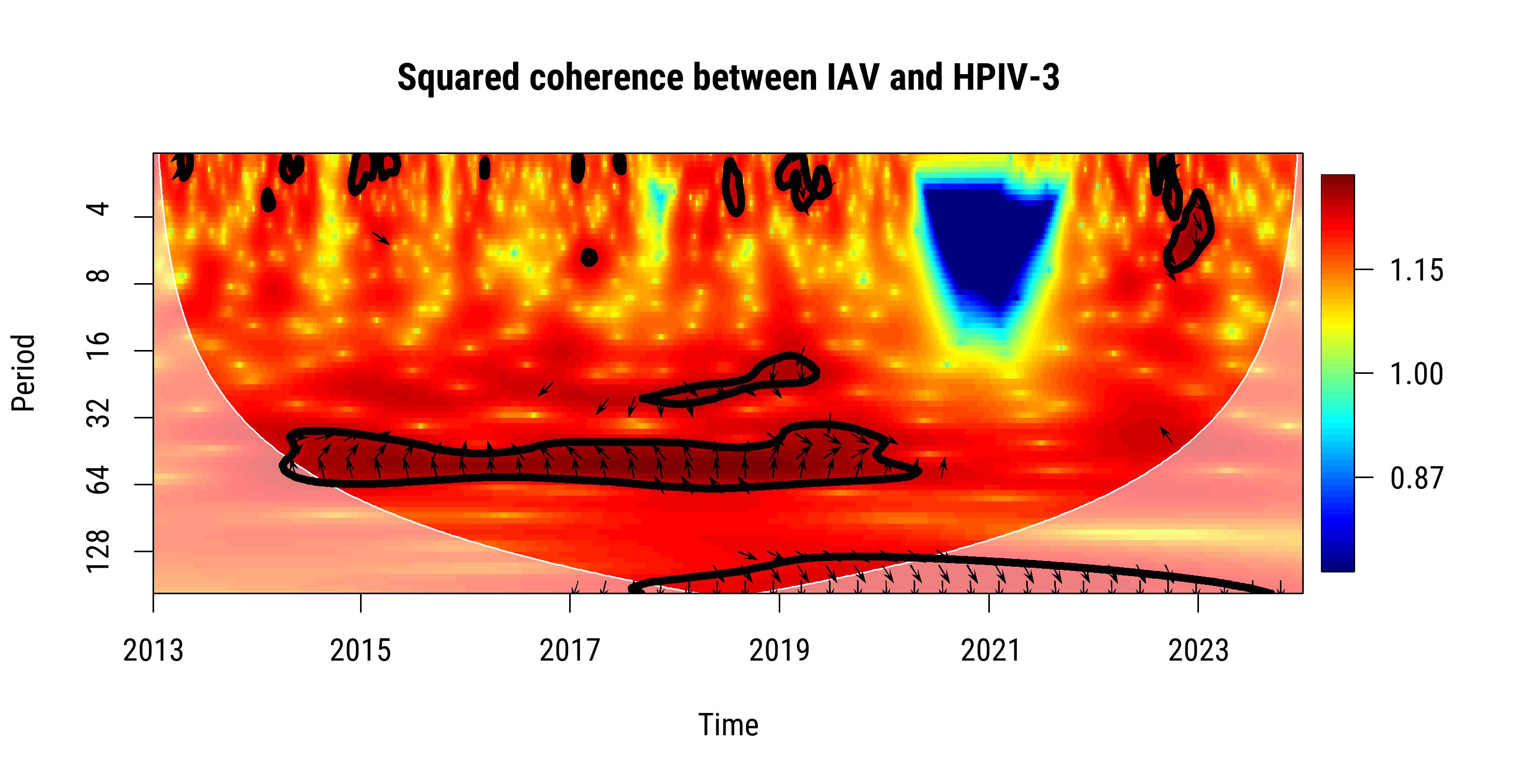** |
| **E.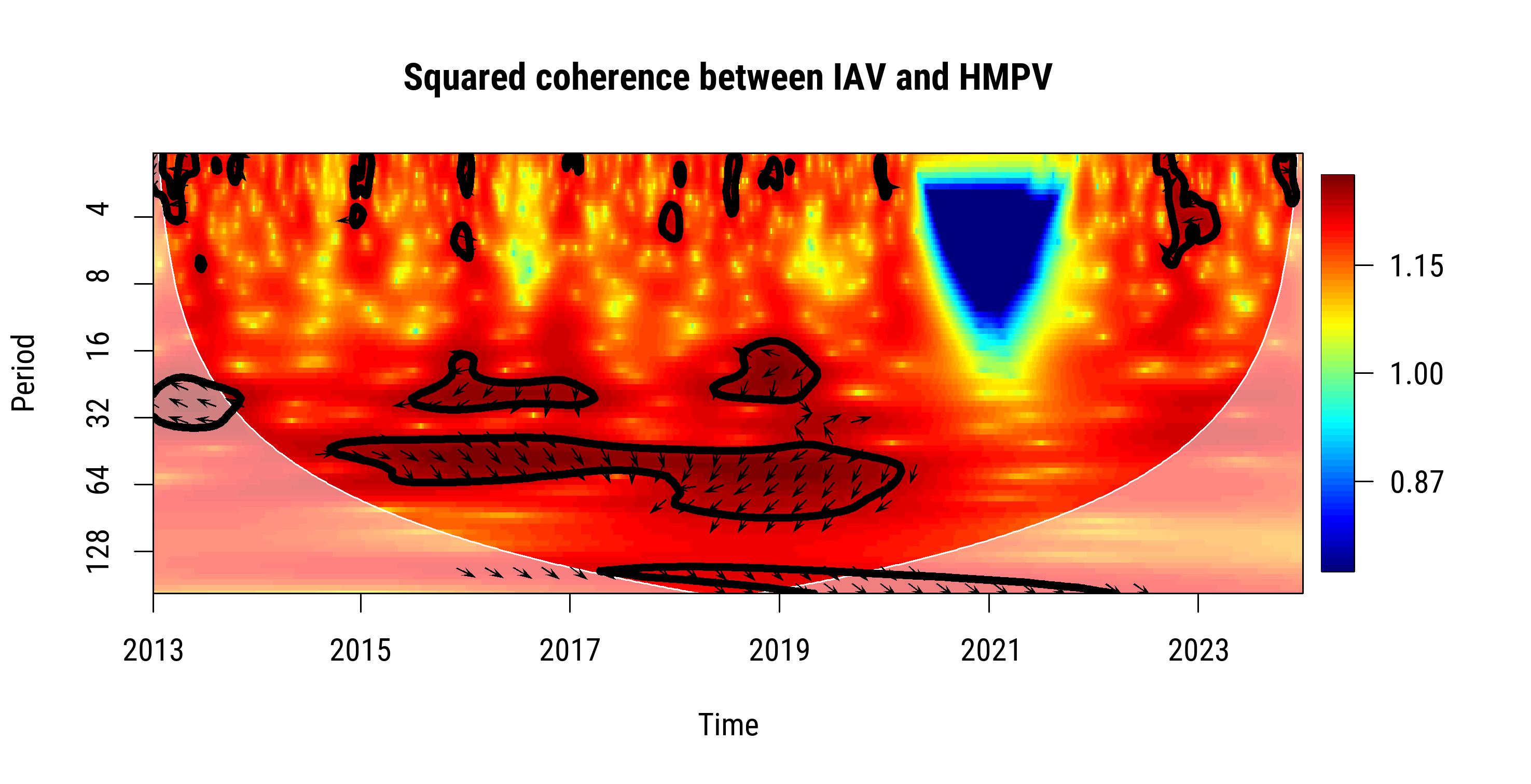** | **F.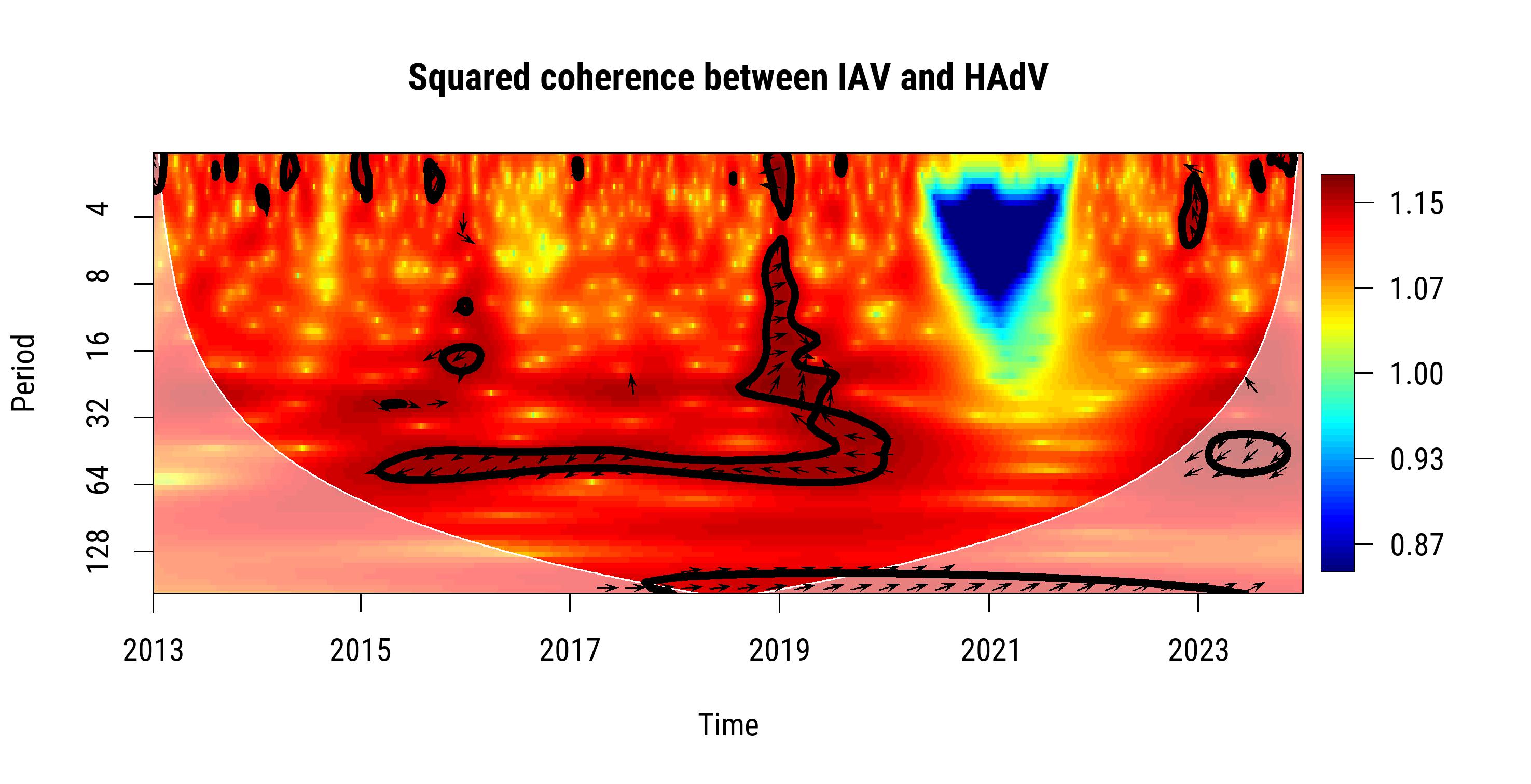** |

| **G.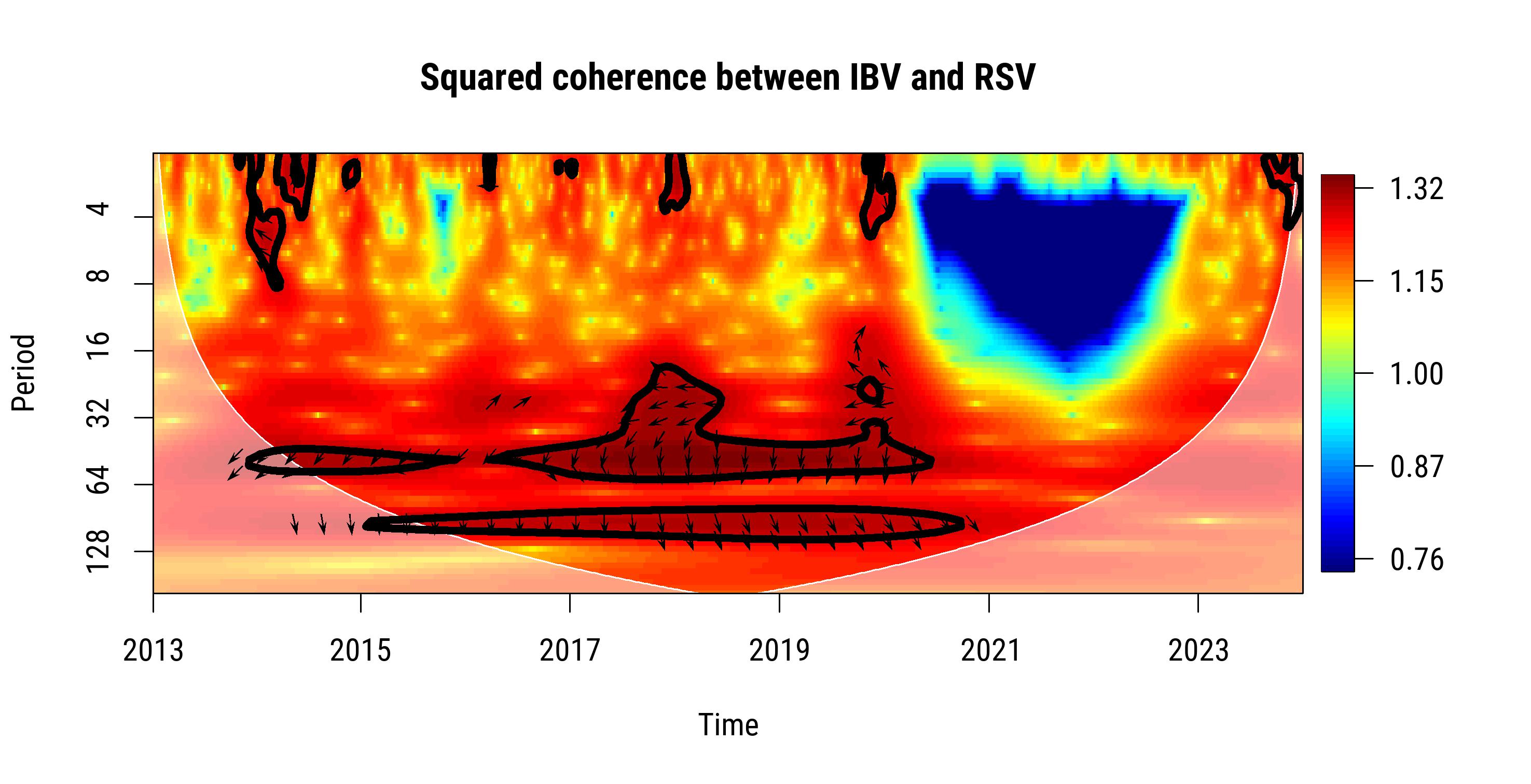** | **H.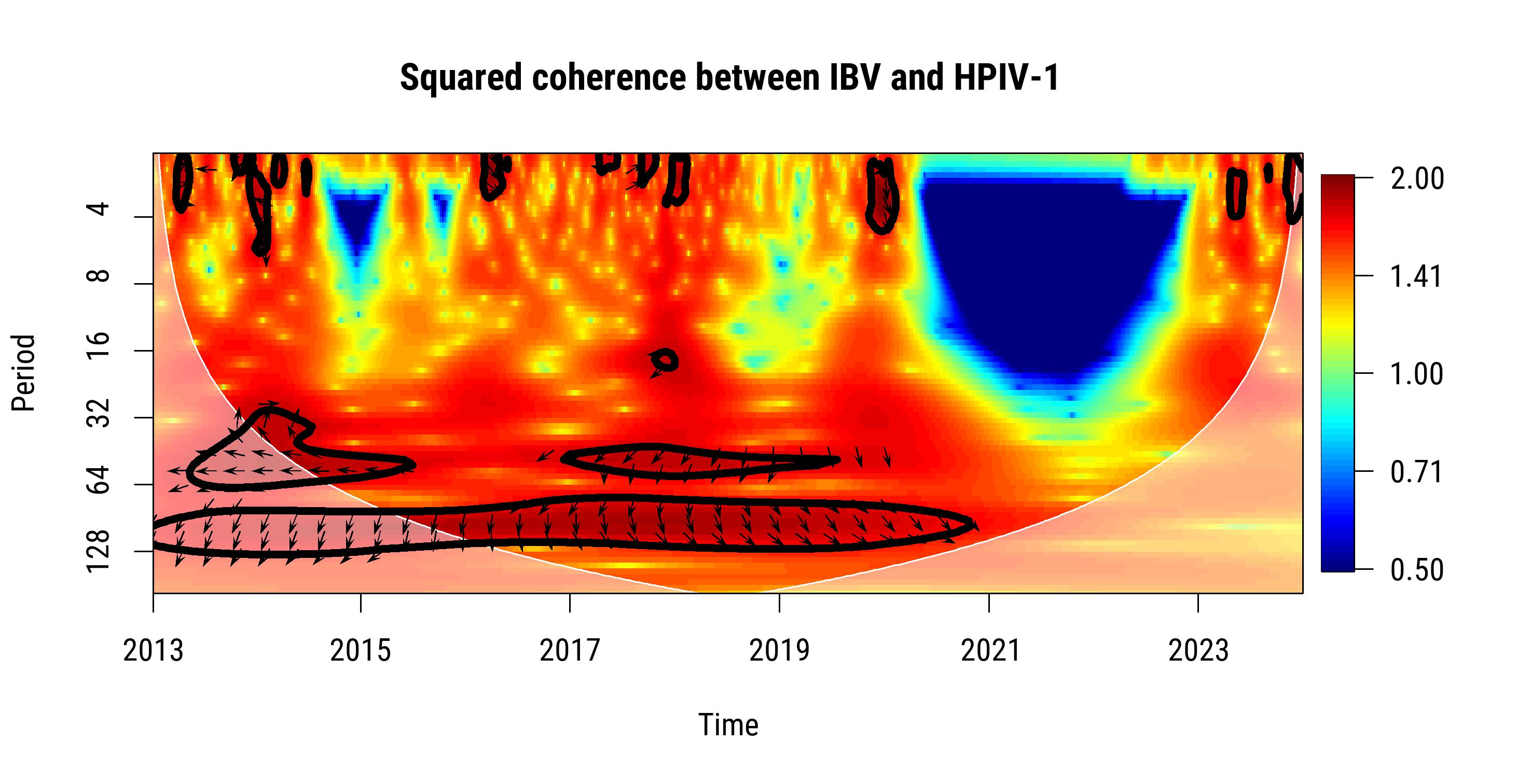** |
| --- | --- |
| **I.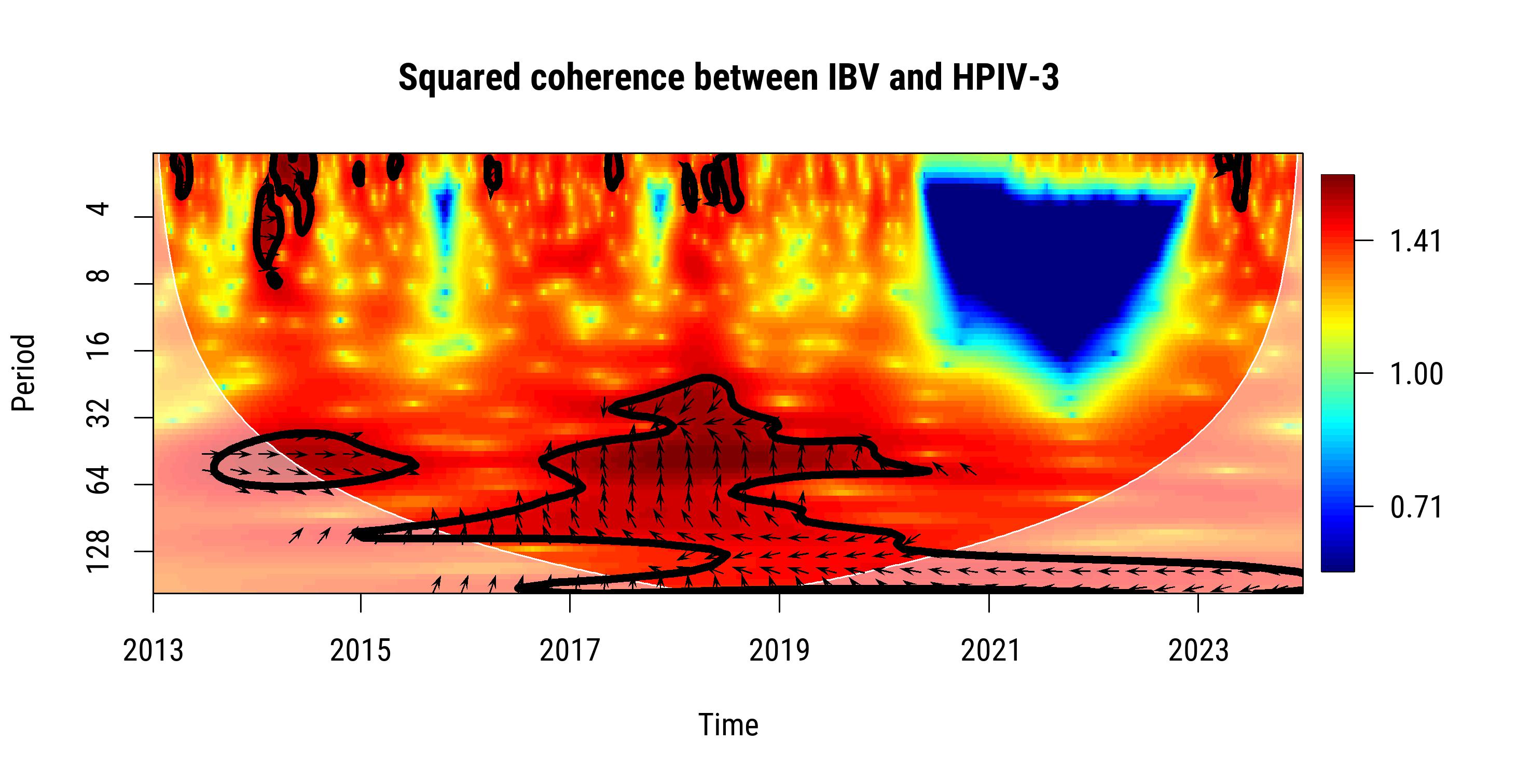** | **J.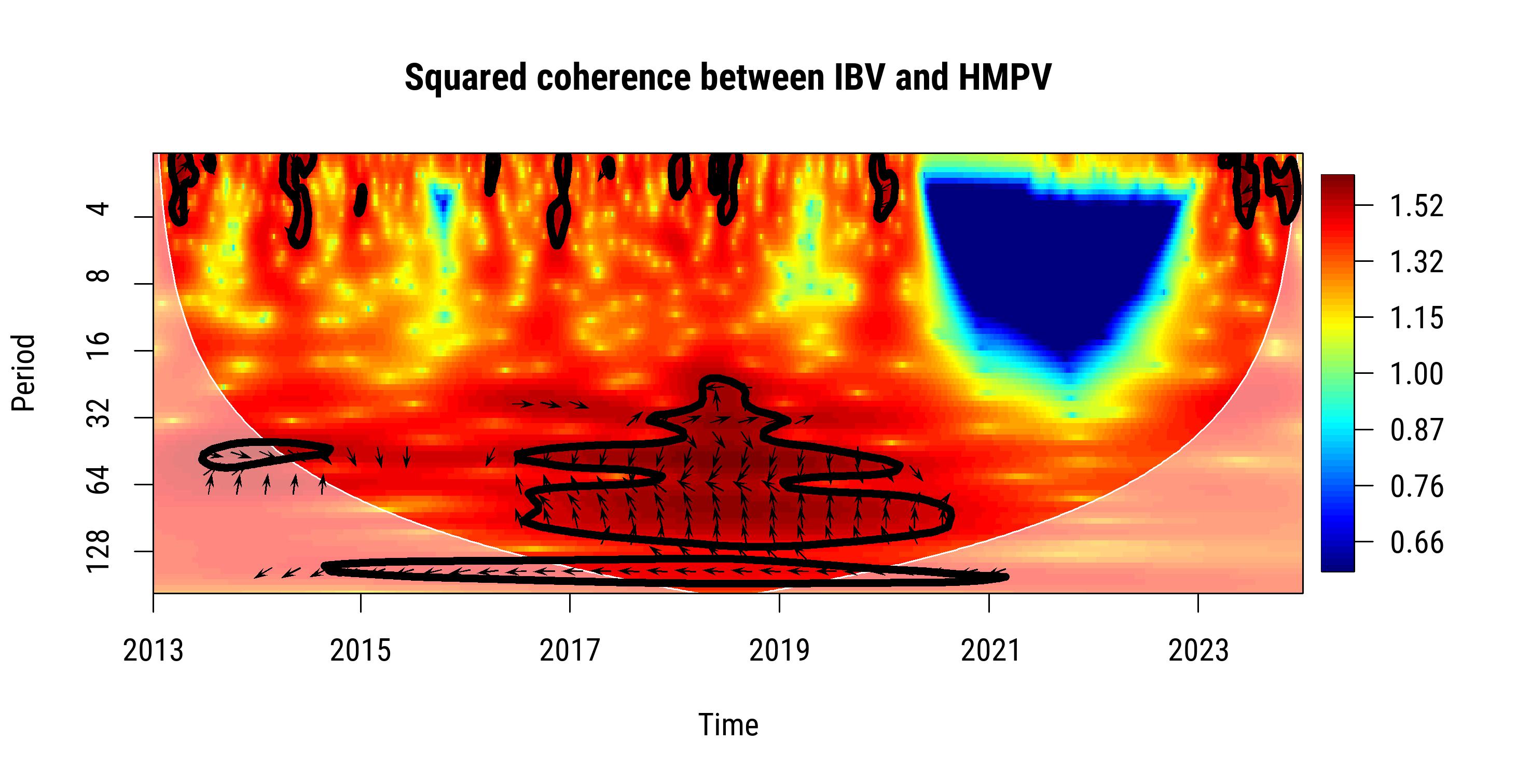** |
| **K.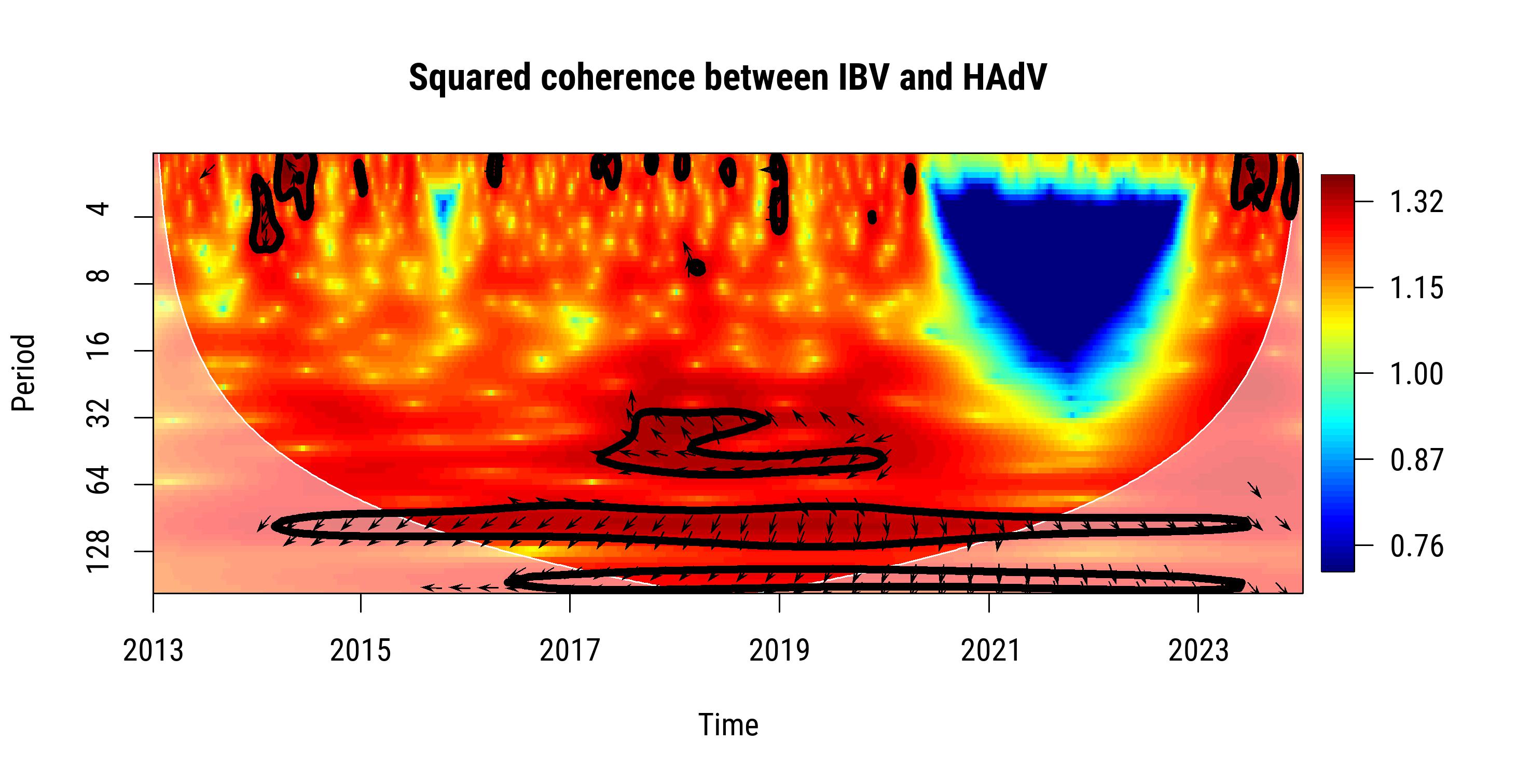** | **L.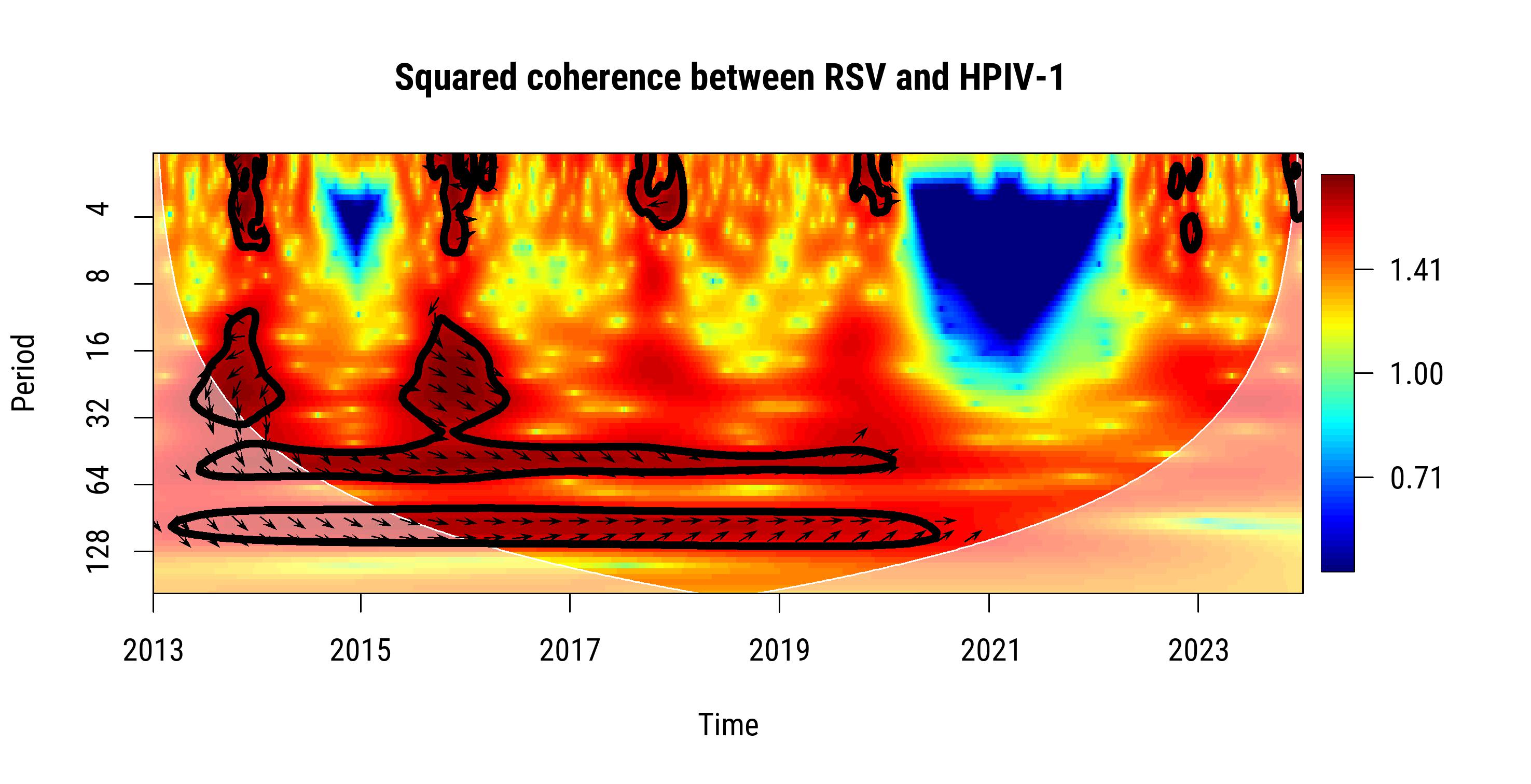** |

| **M.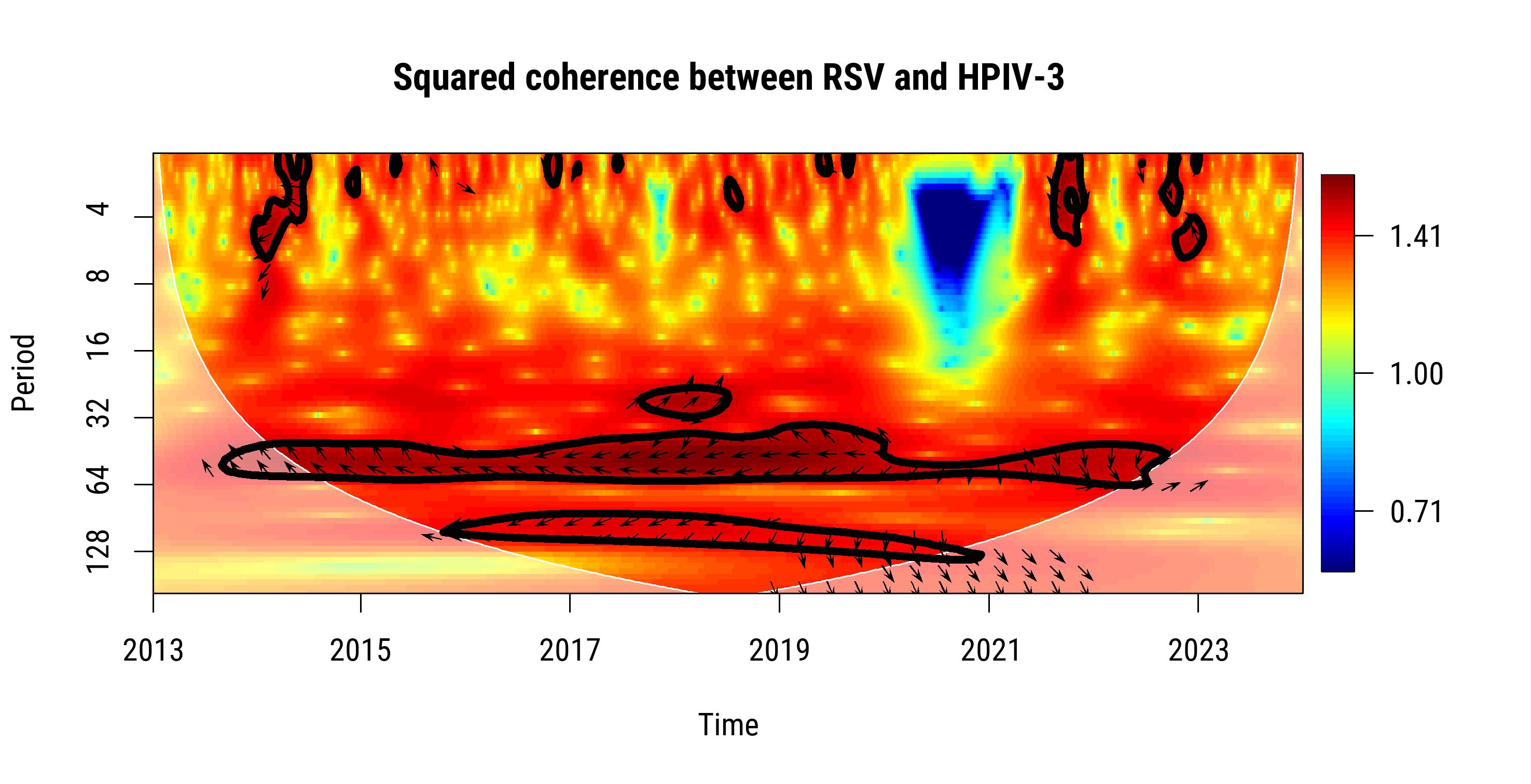** | **N.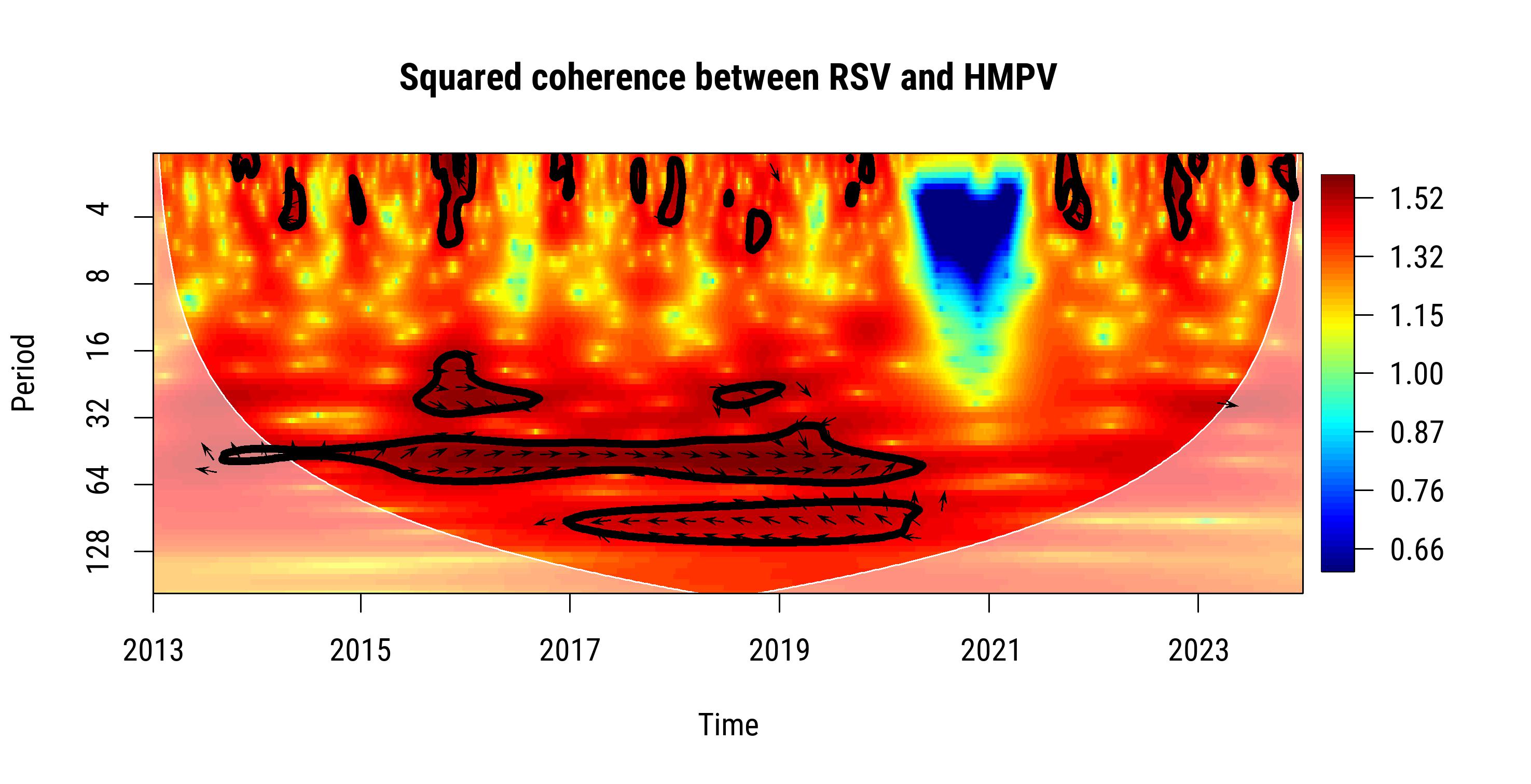** |
| --- | --- |
| **O.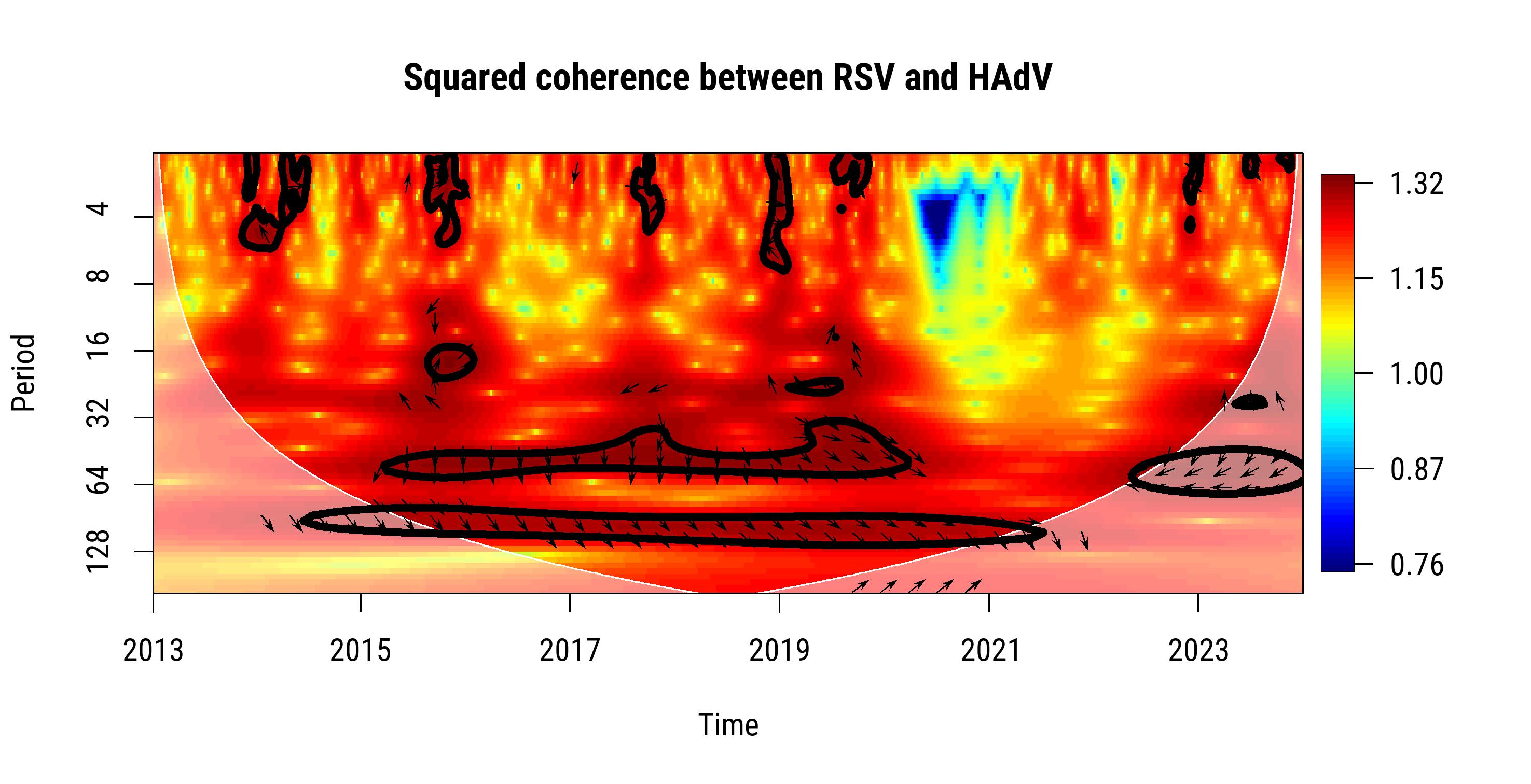** | **P.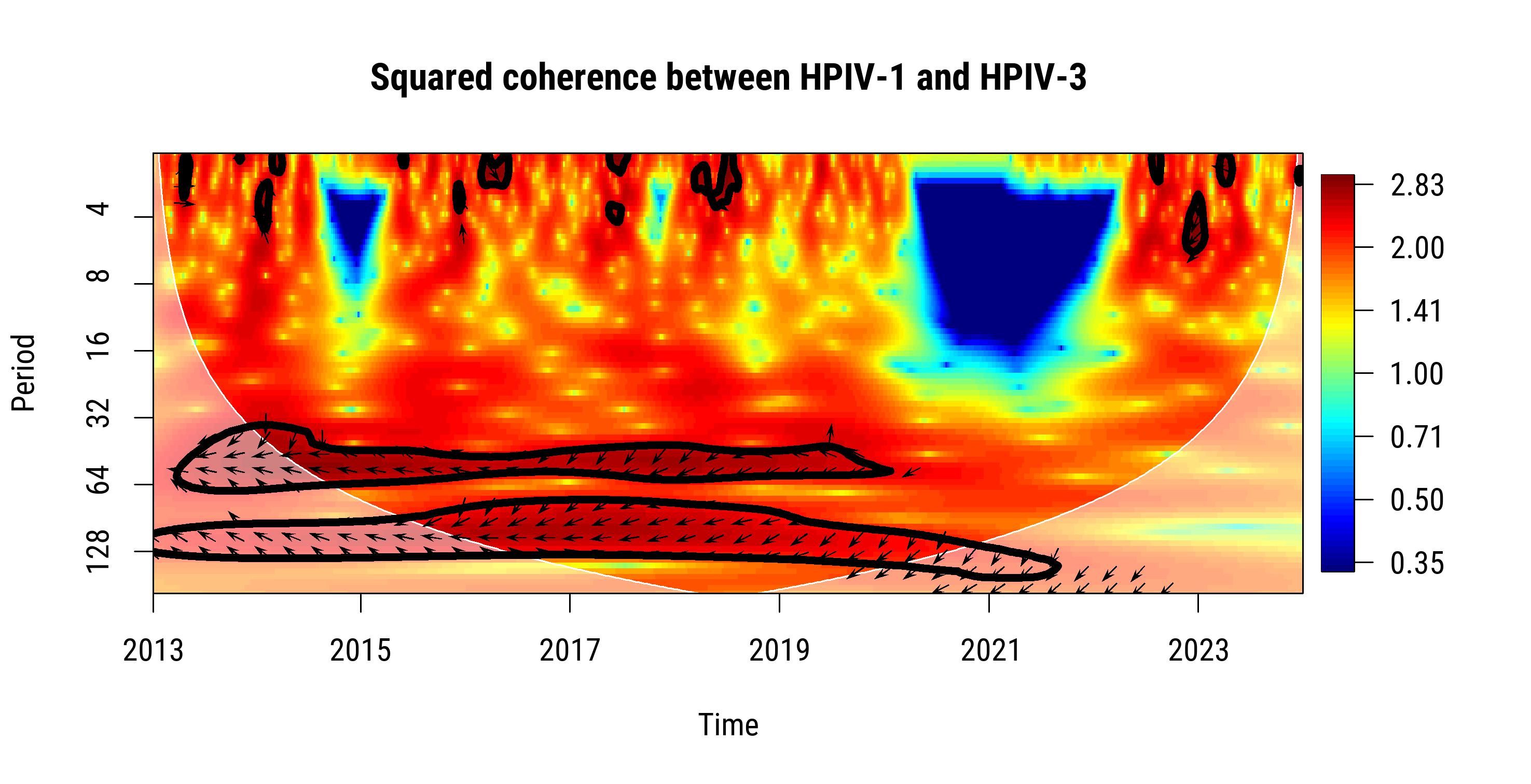** |
| **Q.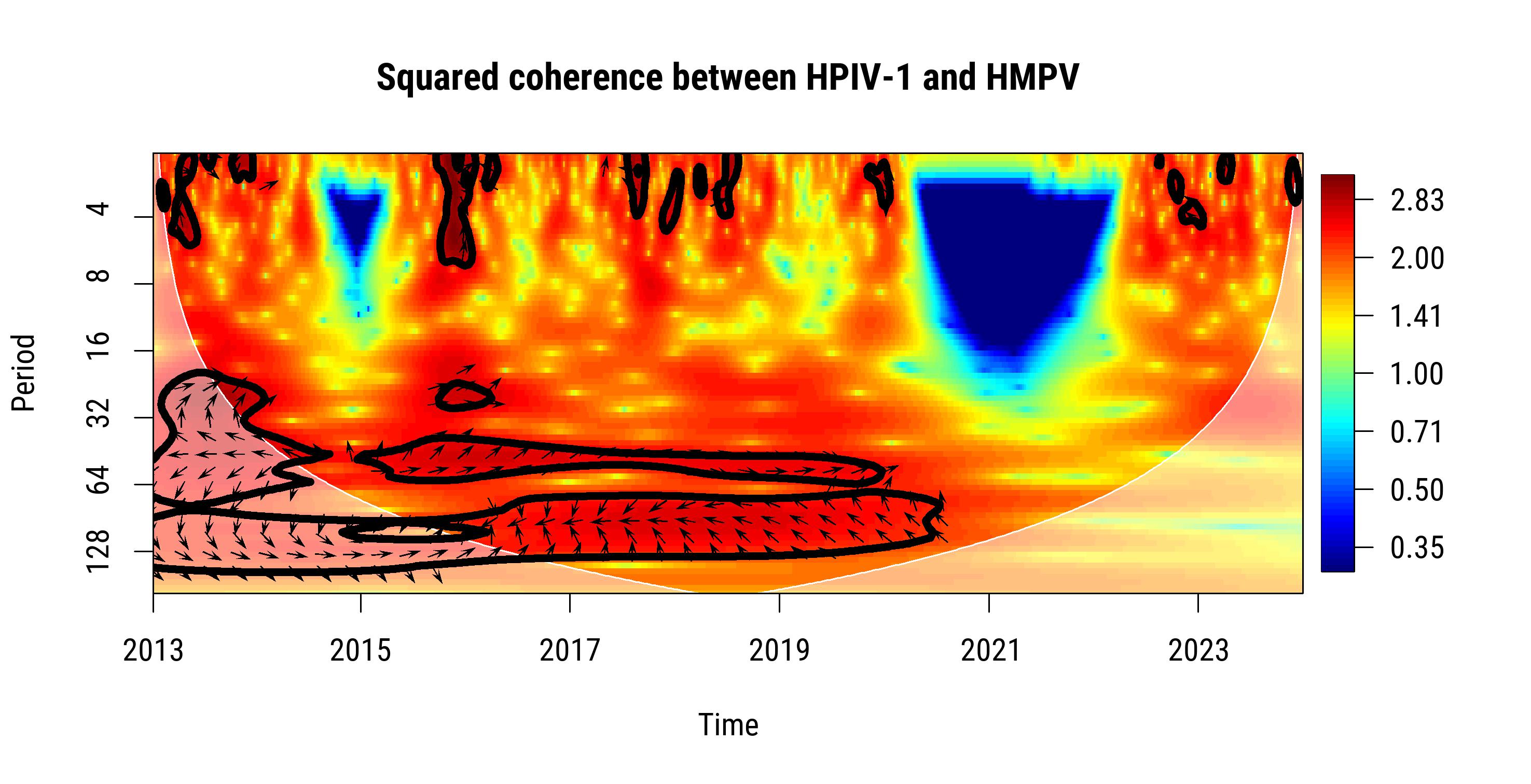** | **R.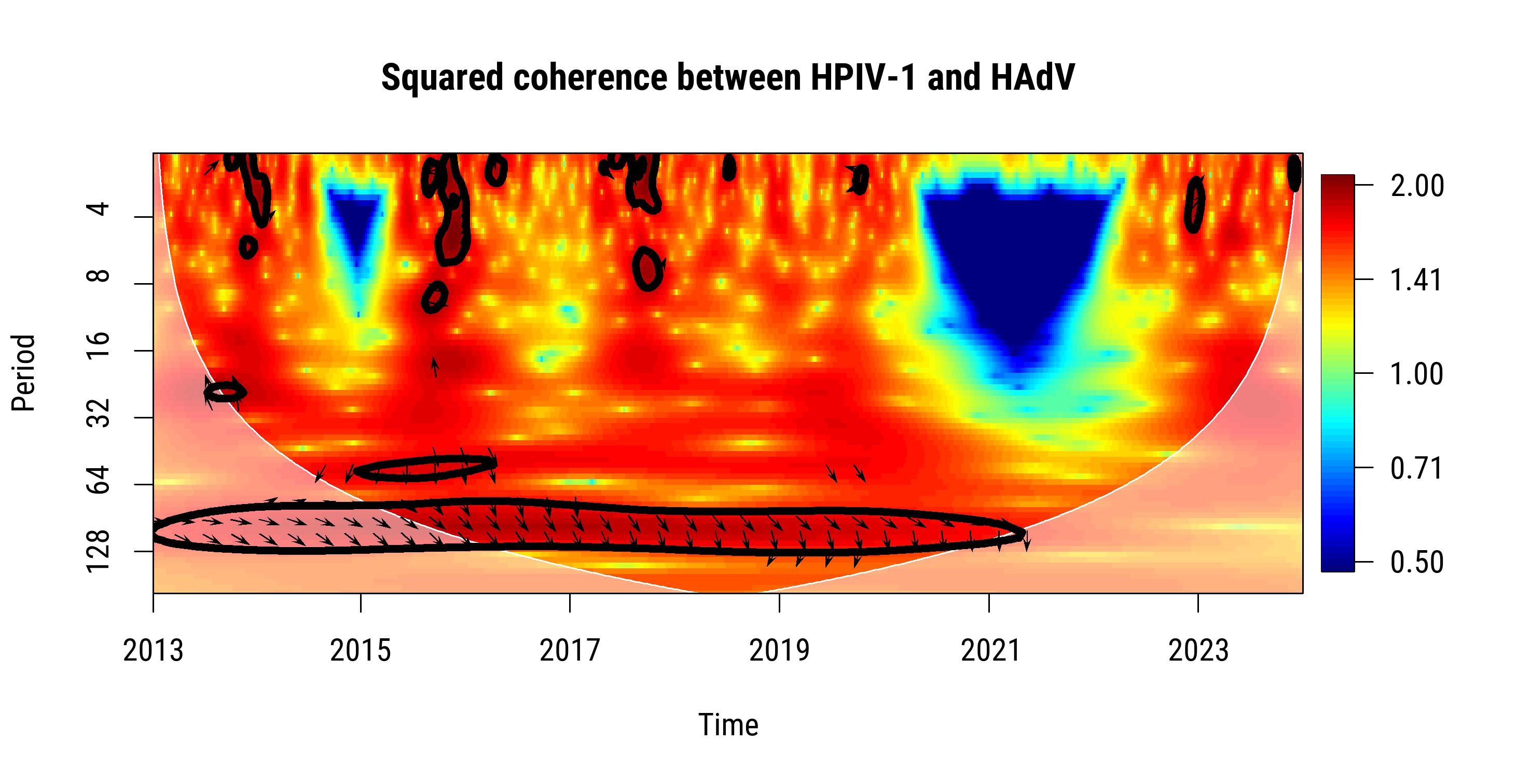** |

| **S.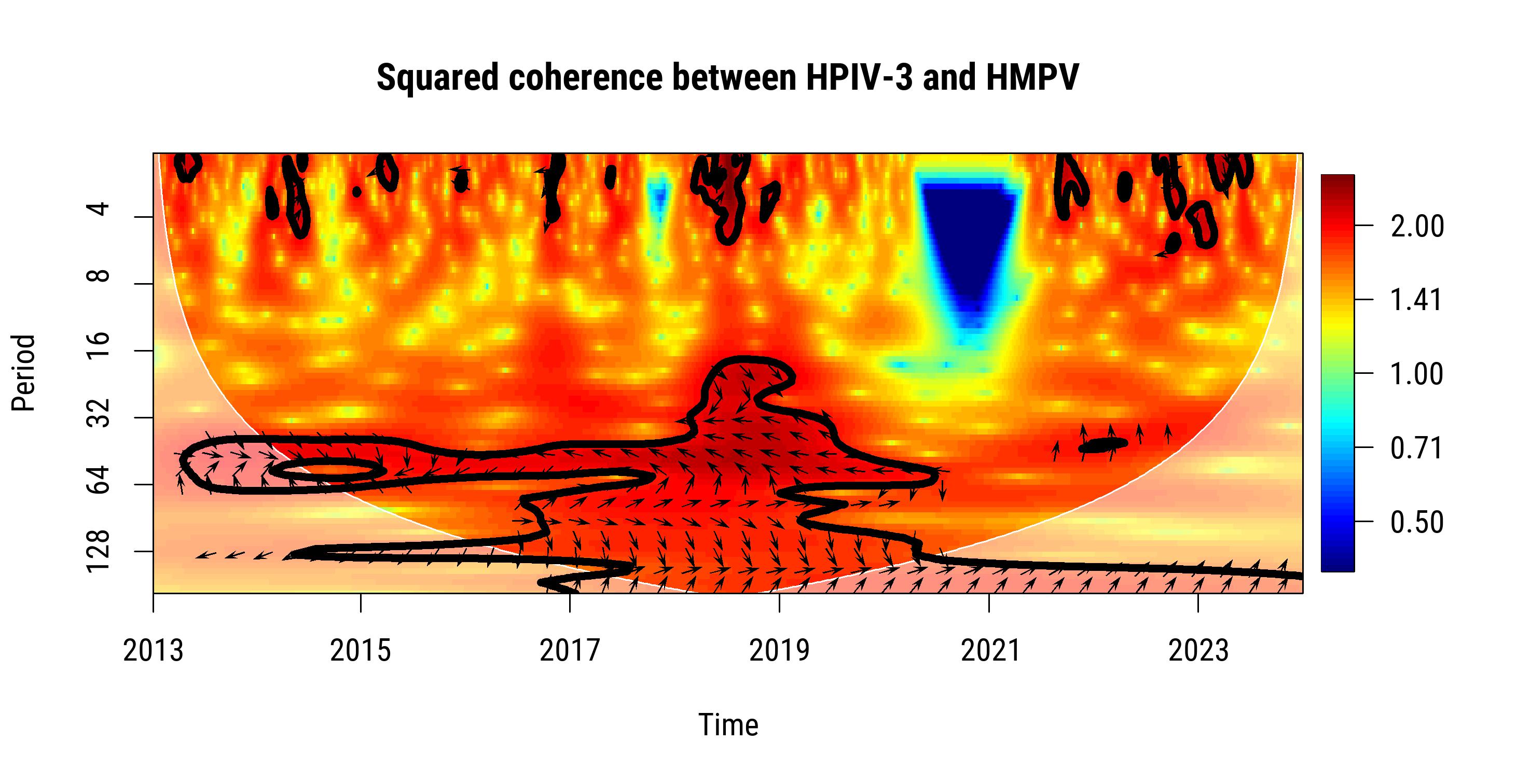** | **T.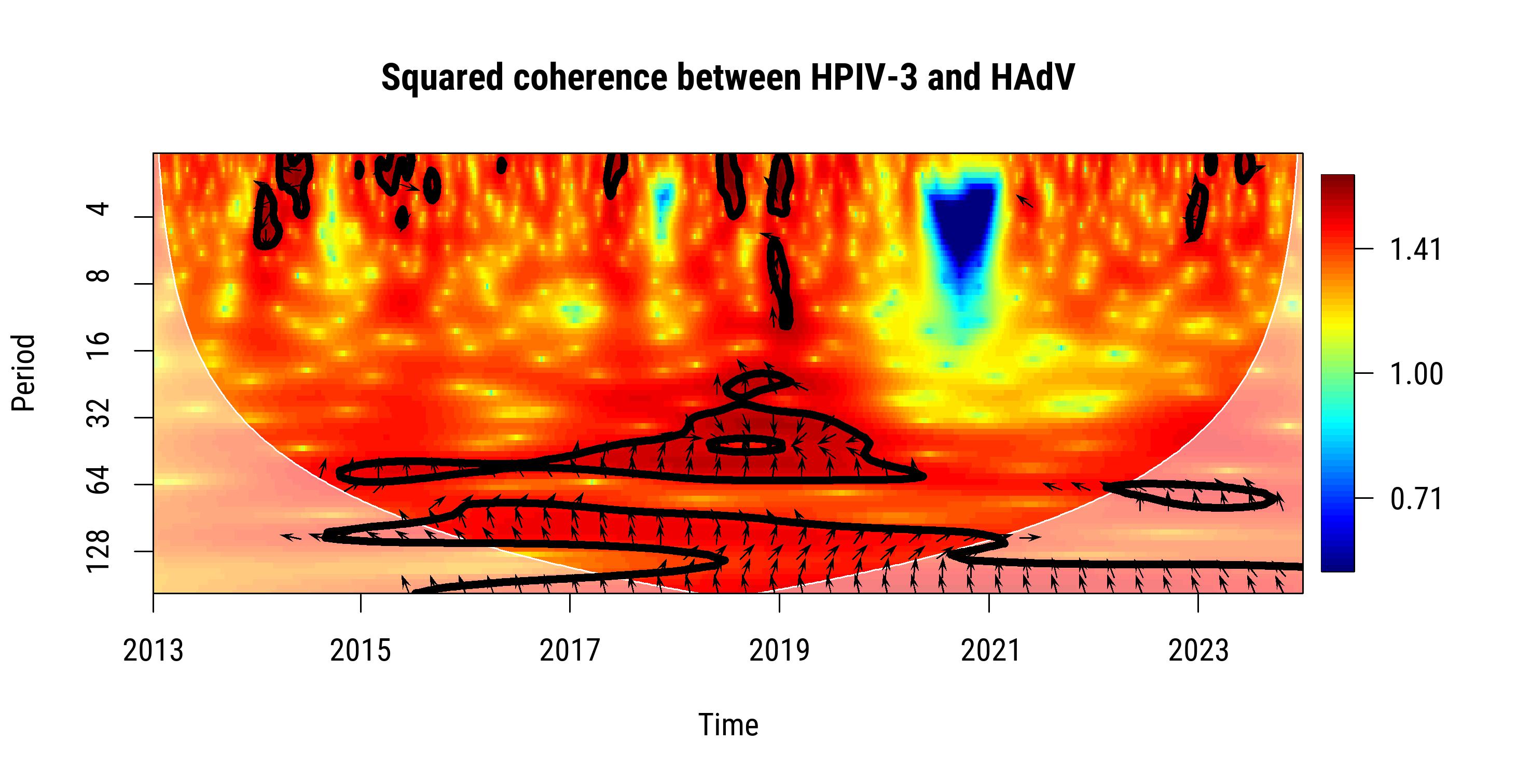** |
| --- | --- |
| **U.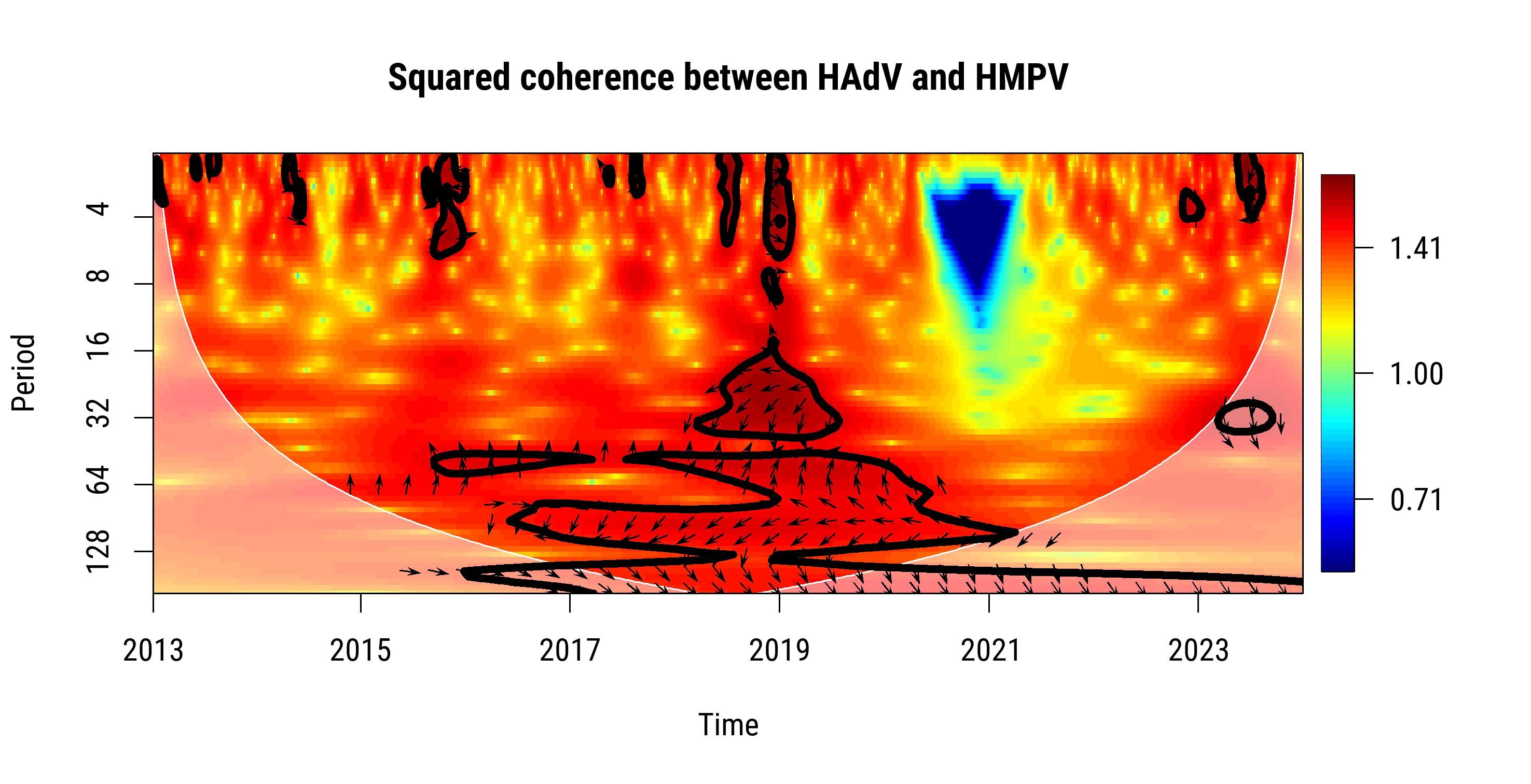** |  |

**Figure S8**. Sub-analysis restricted to children of weighted Pearson’s correlation coefficients of monthly prevalence for each pair of respiratory viruses, adjusted for seasonal and long-term trends, where weights are the numbers of tests administered, Sentinel Enhanced Dengue Surveillance System, Puerto Rico, January 2013 to December 2023. *q*-values are shown which represent statistical evidence adjusted for multiple comparisons by controlling the false discovery rate. Significant correlations (*q* ≤ 0.10) are shown in color. Blue and red indicate positive and negative coefficients, respectively. Example interpretation: The negative correlation between HMPV and HPIV-3 in this sub-analysis suggests that, among children, higher activity of one virus was associated with lower activity of the other during the study period, indicating asynchronous prevalence trends. This is in contrast to the positive correlation between IBV and HMPV, which suggests synchronous co-circulation in this age group. These associations should be interpreted cautiously, as they do not account for potential confounders like shared seasonality or testing patterns.


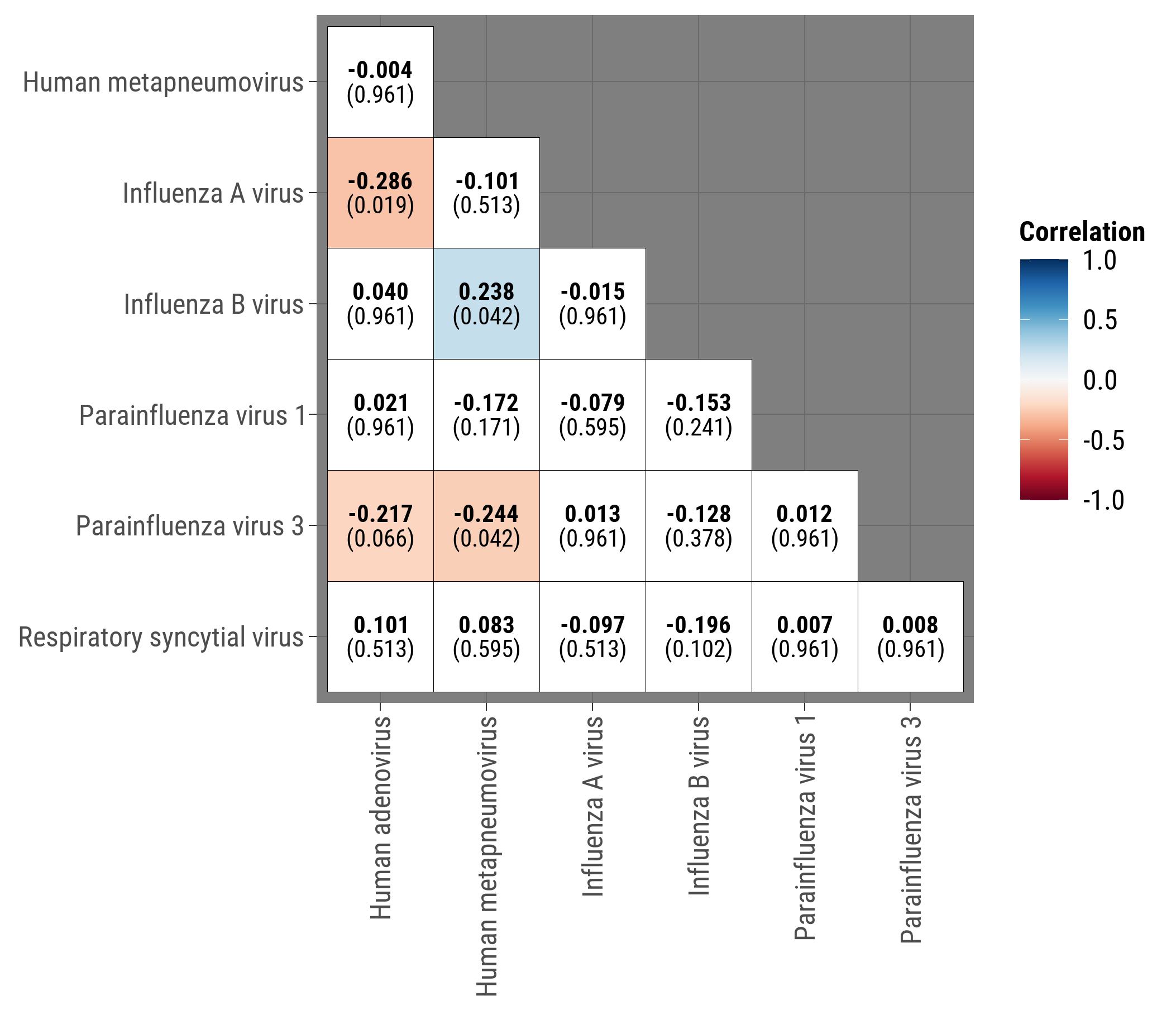


**Figure S9**. Bayesian hierarchical model correlation coefficients adjusting for age, sex, seasonality, changes in testing frequency, and autocorrelation, restricted to children <18 years, Sentinel Enhanced Dengue Surveillance System, Puerto Rico, January 2013 to December 2023. *q*-values are shown which represent statistical evidence adjusted for multiple comparisons by controlling the false discovery rate. Significant correlations (*q* ≤ 0.10) are shown in color. Blue and red indicate positive and negative coefficients, respectively. Example interpretation: For children under 18 years, a positive correlation between RSV and HPIV-3 (*ρ* = 0.66, 95% CrI: 0.41, 0.87, *q* ≤ 0.10) suggests that detection of RSV was strongly associated with an increased likelihood of detecting HPIV-3 during the same period, indicating synchronous circulation of these viruses. This correlation accounts for seasonal trends, testing practices, and demographic factors, highlighting virus co-circulation patterns in this age group. By contrast, no significant negative correlations were observed in this subanalysis.

**
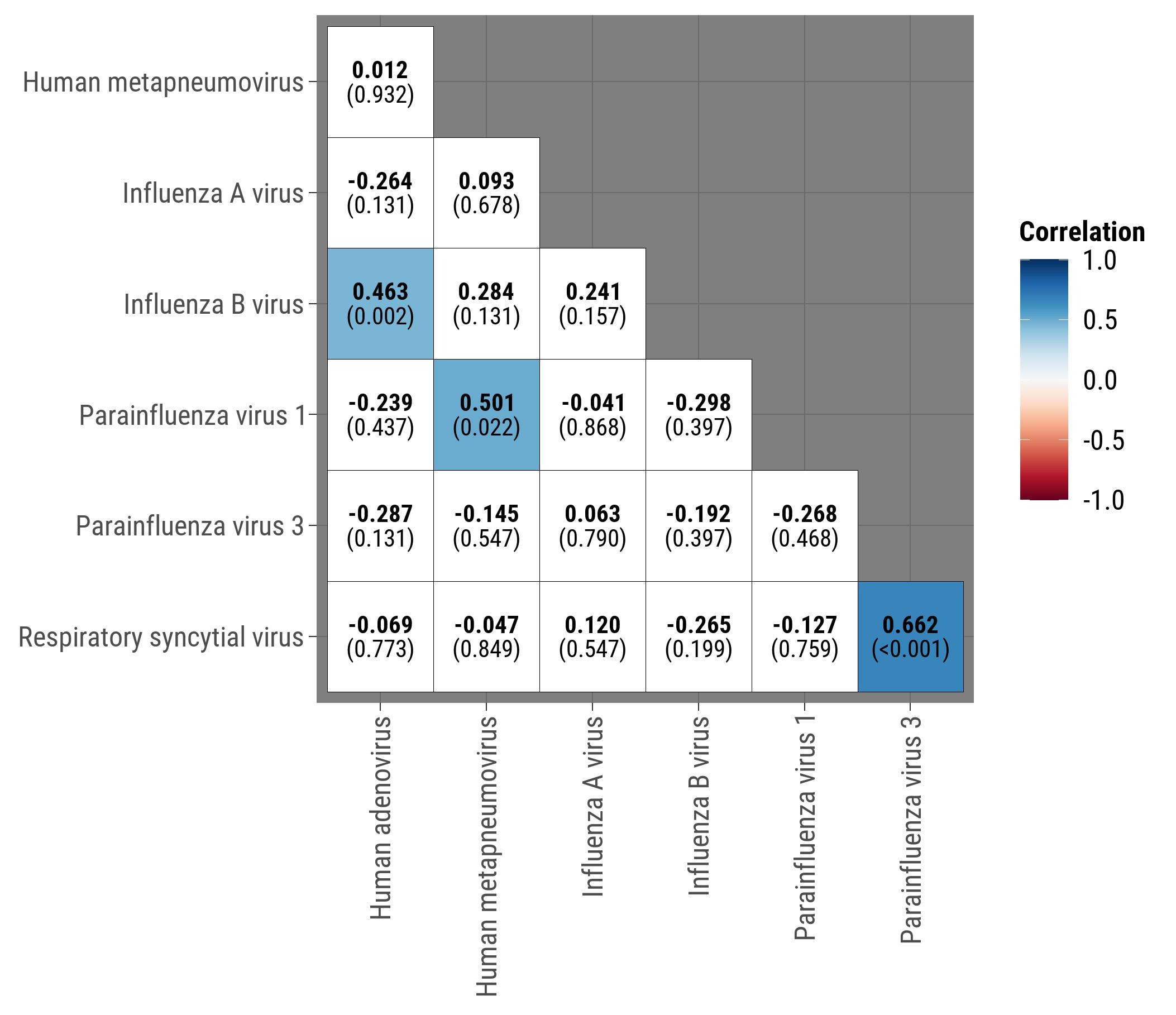
**

| **Table S1**. Mean, standard deviation, and quantiles of the marginal posterior distribution for *ρ*, and convergence diagnostics for Bayesian hierarchical model for the total population, Sentinel Enhanced Dengue Surveillance System, Puerto Rico, January 2013 to December 2023. | | | | | | | | | | | | |
| --- | --- | --- | --- | --- | --- | --- | --- | --- | --- | --- | --- | --- |
| Virus 1 | Virus 2 | Mean | SD | 2.5% | 5.0% | 50.0% | 95.0% | 97.5% | Rhat | n.eff | pD | DIC |
| IAV | IBV | 0.176 | 0.125 | -0.072 | 0.090 | 0.179 | 0.264 | 0.409 | 1.003 | 2000 | 267.9 | 1367.1 |
| IAV | RSV | 0.153 | 0.118 | -0.073 | 0.071 | 0.152 | 0.235 | 0.380 | 1.001 | 4500 | 283.6 | 1448.9 |
| IAV | HAdV | -0.270 | 0.114 | -0.480 | -0.349 | -0.274 | -0.196 | -0.040 | 1.001 | 4500 | 262.3 | 1534.0 |
| IAV | HMPV | 0.213 | 0.115 | -0.020 | 0.135 | 0.216 | 0.293 | 0.428 | 1.003 | 1200 | 263.8 | 1430.9 |
| IAV | HPIV-1 | 0.051 | 0.158 | -0.268 | -0.057 | 0.053 | 0.160 | 0.343 | 1.003 | 1400 | 281.0 | 1308.9 |
| IAV | HPIV-3 | 0.168 | 0.146 | -0.127 | 0.068 | 0.173 | 0.270 | 0.443 | 1.004 | 760 | 283.0 | 1446.1 |
| IBV | RSV | -0.151 | 0.135 | -0.403 | -0.244 | -0.155 | -0.063 | 0.121 | 1.004 | 760 | 292.3 | 1301.6 |
| IBV | HMPV | 0.298 | 0.123 | 0.046 | 0.218 | 0.300 | 0.385 | 0.534 | 1.002 | 1900 | 275.0 | 1290.0 |
| IBV | HAdV | 0.453 | 0.116 | 0.204 | 0.381 | 0.459 | 0.537 | 0.654 | 1.001 | 4500 | 274.7 | 1393.5 |
| IBV | HPIV-1 | -0.164 | 0.194 | -0.535 | -0.300 | -0.169 | -0.033 | 0.217 | 1.006 | 580 | 298.6 | 1171.4 |
| IBV | HPIV-3 | -0.150 | 0.153 | -0.436 | -0.258 | -0.153 | -0.044 | 0.154 | 1.007 | 470 | 299.5 | 1303.5 |
| RSV | HAdV | -0.023 | 0.136 | -0.278 | -0.116 | -0.025 | 0.072 | 0.247 | 1.001 | 3000 | 272.9 | 1447.0 |
| RSV | HPIV-1 | -0.073 | 0.186 | -0.430 | -0.202 | -0.077 | 0.053 | 0.296 | 1.013 | 240 | 295.1 | 1228.7 |
| RSV | HPIV-3 | 0.606 | 0.112 | 0.361 | 0.538 | 0.613 | 0.686 | 0.797 | 1.006 | 540 | 298.8 | 1364.8 |
| RSV | HMPV | 0.109 | 0.149 | -0.197 | 0.011 | 0.114 | 0.211 | 0.384 | 1.004 | 990 | 280.8 | 1355.2 |
| HAdV | HMPV | 0.048 | 0.129 | -0.208 | -0.039 | 0.048 | 0.137 | 0.305 | 1.001 | 4500 | 259.6 | 1439.0 |
| HAdV | HPIV-1 | -0.166 | 0.175 | -0.494 | -0.285 | -0.171 | -0.053 | 0.191 | 1.002 | 1600 | 273.5 | 1310.1 |
| HAdV | HPIV-3 | -0.194 | 0.135 | -0.448 | -0.288 | -0.198 | -0.099 | 0.076 | 1.004 | 770 | 289.6 | 1461.2 |
| HMPV | HPIV-1 | 0.092 | 0.142 | -0.191 | -0.006 | 0.093 | 0.191 | 0.370 | 1.001 | 4400 | 294.0 | 1366.4 |
| HMPV | HPIV-3 | 0.519 | 0.139 | 0.218 | 0.433 | 0.529 | 0.618 | 0.760 | 1.005 | 780 | 270.5 | 1200.8 |
| HPIV-1 | HPIV-3 | -0.243 | 0.198 | -0.610 | -0.377 | -0.252 | -0.110 | 0.159 | 1.006 | 660 | 322.1 | 1251.7 |
| n.eff: effective sample size; DIC: Deviance Information Criterion; pD: effective number of parameters | | | | | | | | | | | | |

| **Table S2**. Mean, standard deviation, and quantiles of the marginal posterior distribution for *ρ*, and convergence diagnostics for Bayesian hierarchical model restricted to children <18 years old, Sentinel Enhanced Dengue Surveillance System, Puerto Rico, January 2013 to December 2023. | | | | | | | | | | | | |
| --- | --- | --- | --- | --- | --- | --- | --- | --- | --- | --- | --- | --- |
| Virus 1 | Virus 2 | Mean | SD | 2.5% | 5.0% | 50.0% | 95.0% | 97.5% | Rhat | n.eff | pD | DIC |
| IAV | IBV | 0.176 | 0.125 | -0.072 | 0.090 | 0.179 | 0.264 | 0.409 | 1.003 | 2000 | 267.9 | 1367.1 |
| IAV | RSV | 0.153 | 0.118 | -0.073 | 0.071 | 0.152 | 0.235 | 0.380 | 1.001 | 4500 | 283.6 | 1448.9 |
| IAV | HAdV | -0.270 | 0.114 | -0.480 | -0.349 | -0.274 | -0.196 | -0.040 | 1.001 | 4500 | 262.3 | 1534.0 |
| IAV | HMPV | 0.213 | 0.115 | -0.020 | 0.135 | 0.216 | 0.293 | 0.428 | 1.003 | 1200 | 263.8 | 1430.9 |
| IAV | HPIV-1 | 0.051 | 0.158 | -0.268 | -0.057 | 0.053 | 0.160 | 0.343 | 1.003 | 1400 | 281.0 | 1308.9 |
| IAV | HPIV-3 | 0.168 | 0.146 | -0.127 | 0.068 | 0.173 | 0.270 | 0.443 | 1.004 | 760 | 283.0 | 1446.1 |
| IBV | RSV | -0.151 | 0.135 | -0.403 | -0.244 | -0.155 | -0.063 | 0.121 | 1.004 | 760 | 292.3 | 1301.6 |
| IBV | HMPV | 0.298 | 0.123 | 0.046 | 0.218 | 0.300 | 0.385 | 0.534 | 1.002 | 1900 | 275.0 | 1290.0 |
| IBV | HAdV | 0.453 | 0.116 | 0.204 | 0.381 | 0.459 | 0.537 | 0.654 | 1.001 | 4500 | 274.7 | 1393.5 |
| IBV | HPIV-1 | -0.164 | 0.194 | -0.535 | -0.300 | -0.169 | -0.033 | 0.217 | 1.006 | 580 | 298.6 | 1171.4 |
| IBV | HPIV-3 | -0.150 | 0.153 | -0.436 | -0.258 | -0.153 | -0.044 | 0.154 | 1.007 | 470 | 299.5 | 1303.5 |
| RSV | HAdV | -0.023 | 0.136 | -0.278 | -0.116 | -0.025 | 0.072 | 0.247 | 1.001 | 3000 | 272.9 | 1447.0 |
| RSV | HPIV-1 | -0.073 | 0.186 | -0.430 | -0.202 | -0.077 | 0.053 | 0.296 | 1.013 | 240 | 295.1 | 1228.7 |
| RSV | HPIV-3 | 0.606 | 0.112 | 0.361 | 0.538 | 0.613 | 0.686 | 0.797 | 1.006 | 540 | 298.8 | 1364.8 |
| RSV | HMPV | 0.109 | 0.149 | -0.197 | 0.011 | 0.114 | 0.211 | 0.384 | 1.004 | 990 | 280.8 | 1355.2 |
| HAdV | HMPV | 0.048 | 0.129 | -0.208 | -0.039 | 0.048 | 0.137 | 0.305 | 1.001 | 4500 | 259.6 | 1439.0 |
| HAdV | HPIV-1 | -0.166 | 0.175 | -0.494 | -0.285 | -0.171 | -0.053 | 0.191 | 1.002 | 1600 | 273.5 | 1310.1 |
| HAdV | HPIV-3 | -0.194 | 0.135 | -0.448 | -0.288 | -0.198 | -0.099 | 0.076 | 1.004 | 770 | 289.6 | 1461.2 |
| HMPV | HPIV-1 | 0.092 | 0.142 | -0.191 | -0.006 | 0.093 | 0.191 | 0.370 | 1.001 | 4400 | 294.0 | 1366.4 |
| HMPV | HPIV-3 | 0.519 | 0.139 | 0.218 | 0.433 | 0.529 | 0.618 | 0.760 | 1.005 | 780 | 270.5 | 1200.8 |
| HPIV-1 | HPIV-3 | -0.243 | 0.198 | -0.610 | -0.377 | -0.252 | -0.110 | 0.159 | 1.006 | 660 | 322.1 | 1251.7 |
| n.eff: effective sample size; DIC: Deviance Information Criterion; pD: effective number of parameters | | | | | | | | | | | | |

| **Table S3**. Mean, standard deviation, and quantiles of the marginal posterior distribution for *ρ*, and convergence diagnostics for Bayesian hierarchical model for the total population restricted to the pre-COVID-19 pandemic period, Sentinel Enhanced Dengue Surveillance System, Puerto Rico, January 2013 to December 2019. | | | | | | | | | | | | |
| --- | --- | --- | --- | --- | --- | --- | --- | --- | --- | --- | --- | --- |
| Virus 1 | Virus 2 | Mean | SD | 2.5% | 5.0% | 50.0% | 95.0% | 97.5% | Rhat | n.eff | pD | DIC |
| IAV | IBV | 0.000 | 0.142 | -0.279 | -0.098 | 0.000 | 0.097 | 0.277 | 1.003 | 2200 | 179.7 | 1003.8 |
| IAV | RSV | 0.110 | 0.140 | -0.172 | 0.018 | 0.115 | 0.207 | 0.368 | 1.001 | 5000 | 171.7 | 994.2 |
| IAV | HAdV | -0.425 | 0.129 | -0.657 | -0.515 | -0.435 | -0.343 | -0.143 | 1.002 | 2500 | 181.9 | 1089.8 |
| IAV | HMPV | -0.048 | 0.142 | -0.319 | -0.146 | -0.050 | 0.050 | 0.234 | 1.003 | 1800 | 165.0 | 980.8 |
| IAV | HPIV-1 | -0.118 | 0.239 | -0.546 | -0.288 | -0.130 | 0.044 | 0.366 | 1.003 | 2200 | 136.2 | 876.4 |
| IAV | HPIV-3 | 0.021 | 0.203 | -0.375 | -0.120 | 0.022 | 0.161 | 0.418 | 1.002 | 3500 | 168.0 | 974.6 |
| IBV | RSV | -0.320 | 0.166 | -0.629 | -0.439 | -0.324 | -0.208 | 0.018 | 1.002 | 2800 | 177.1 | 930.1 |
| IBV | HMPV | 0.270 | 0.151 | -0.039 | 0.169 | 0.277 | 0.377 | 0.547 | 1.005 | 1200 | 174.1 | 918.4 |
| IBV | HAdV | -0.033 | 0.168 | -0.359 | -0.151 | -0.035 | 0.083 | 0.298 | 1.002 | 2200 | 170.4 | 1000.7 |
| IBV | HPIV-1 | -0.218 | 0.298 | -0.737 | -0.435 | -0.234 | -0.021 | 0.409 | 1.013 | 450 | 139.9 | 807.9 |
| IBV | HPIV-3 | -0.218 | 0.207 | -0.590 | -0.364 | -0.230 | -0.078 | 0.204 | 1.003 | 2000 | 176.0 | 908.6 |
| RSV | HAdV | 0.284 | 0.155 | -0.035 | 0.180 | 0.292 | 0.394 | 0.559 | 1.006 | 910 | 179.6 | 1007.1 |
| RSV | HPIV-1 | -0.559 | 0.245 | -0.892 | -0.750 | -0.605 | -0.412 | 0.038 | 1.020 | 280 | 124.5 | 792.5 |
| RSV | HPIV-3 | 0.052 | 0.257 | -0.477 | -0.123 | 0.062 | 0.234 | 0.525 | 1.003 | 1800 | 171.8 | 904.4 |
| RSV | HMPV | 0.018 | 0.183 | -0.339 | -0.109 | 0.018 | 0.144 | 0.374 | 1.002 | 2300 | 170.9 | 913.0 |
| HAdV | HMPV | -0.150 | 0.162 | -0.465 | -0.263 | -0.156 | -0.042 | 0.176 | 1.001 | 5000 | 164.9 | 986.9 |
| HAdV | HPIV-1 | 0.032 | 0.306 | -0.548 | -0.188 | 0.032 | 0.247 | 0.616 | 1.007 | 840 | 139.4 | 885.6 |
| HAdV | HPIV-3 | -0.039 | 0.254 | -0.533 | -0.218 | -0.040 | 0.136 | 0.461 | 1.004 | 1300 | 188.2 | 1001.5 |
| HMPV | HPIV-1 | 0.127 | 0.223 | -0.319 | -0.021 | 0.134 | 0.283 | 0.549 | 1.002 | 2600 | 167.4 | 891.0 |
| HMPV | HPIV-3 | 0.351 | 0.273 | -0.241 | 0.174 | 0.382 | 0.556 | 0.790 | 1.011 | 550 | 132.0 | 789.4 |
| HPIV-1 | HPIV-3 | -0.152 | 0.452 | -0.838 | -0.534 | -0.205 | 0.207 | 0.720 | 1.038 | 160 | 123.0 | 771.8 |
| n.eff: effective sample size; DIC: Deviance Information Criterion; pD: effective number of parameters | | | | | | | | | | | | |
